# A Combined Experimental/Computational Study of Dicationic Ionic Liquids with Bromide and Tungstate Anions

**DOI:** 10.3390/molecules29092131

**Published:** 2024-05-03

**Authors:** Guelber Cardoso Gomes, Claudio Ferdeghini, Luca Guglielmero, Felicia D’Andrea, Lorenzo Guazzelli, Andrea Mezzetta, Christian Silvio Pomelli

**Affiliations:** 1 Department of Pharmacy, University of Pisa, Via Bonanno 33, 56126 Pisa, Italy; guelbercardoso@gmail.com (G.C.G.); claudioferdeghini@gmail.com (C.F.); felicia.dandrea@unipi.it (F.D.); lorenzo.guazzelli@unipi.it (L.G.); andrea.mezzetta@unipi.it (A.M.); 2 Classe di Scienze, Scuola Normale Superiore, Piazza dei Cavalieri 7, 56126 Pisa, Italy

**Keywords:** ionic liquids, tungstate ion, DFT, catalysis, dicationic ionic liquids

## Abstract

A panel of dicationic ionic liquids (DILs) with different rigid xylyl (ortho, meta, para) spacers and different anions (bromide and tungstate) has been synthetised and characterised through different experimental and computational techniques. Differences and analogies between the systems are analysed using information derived from their DFT structures, semiempirical dynamics, thermal behaviour, and catalytic properties versus the well-known reaction of CO_2_ added to epichlorohydrin. A comparison between the proposed systems and some analogues that present non-rigid spacers shows the key effect displayed by structure rigidity on their characteristics. The results show an interesting correlation between structure, flexibility, properties, and catalytic activity.

## 1. Introduction

**Figure 1:**
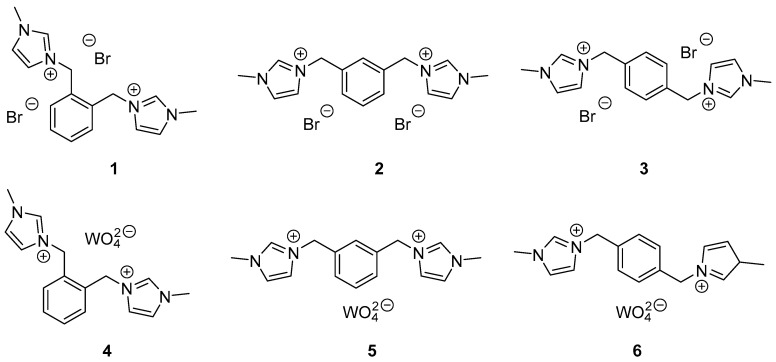
Bromide and tungstate DILs synthesised and studied in this paper.

Academic interest in the study of ionic liquids (ILs) has grown constantly during recent decades [1]. The peculiar set of physical–chemical properties displayed by ILs, arising from their structure composed only of ions, has made them valuable systems in a wide variety of applications, including dissolution, fractionation, and valorisation of biomasses [2,3,4,5,6]; organic synthesis and catalysis [7,8,9]; electrochemistry; energy storage devices [10,11,12,13,14]; and, more recently, biological and pharmaceutical applications [15,16]. Furthermore, ILs typically display negligible vapour pressure, high thermal stability, and low flammability, which have gained this neoteric class of solvents a generally accepted reputation for being relatively green media [17,18,19,20,21,22]. Finally, the great number of possible IL structures offers the possibility of designing and preparing task-specific systems characterized by finely tuned properties by selecting an appropriate couple of ions [23]. Dicationic ionic liquids (DILs), a class of ILs that display a positive ion consisting of two cationic moieties covalently connected to each other, can offer an even higher structural variability [24,25,26,27,28]. The possibility of tuning the physical–chemical properties of DILs by changing the type and the length of the spacer chain has been assessed and explored by various studies in the literature [25,28,29,30,31,32]. DILs can be useful also as charged tags for the study of reactivity in ILs through use of ESI mass techniques [33,34]. The nature of the spacer (length, type of chain) has been reported to have a strong effect on the thermal stability, melting point, electrochemical window, and solubility characteristics of DILs [25,29,31,32,35,36,37]. The possibility of undergoing specific thermally induced rearrangements has also been pointed out to be directly determined by the length of the spacer [38,39]. Furthermore, the linker chain structure was also identified to be an important factor in the determination of the catalytic activity of imidazolium DILs toward the reaction of cyclocarbonation of epoxides [36,40,41]. The use of ILs for the dissolution of metal ions and complexes has already gained a certain popularity in research fields such as electrochemistry and metal recovery from waste materials [42,43,44,45,46,47]. In parallel, the inclusion of catalytically or electrochemically active metal-based systems in ILs structures is attracting growing interest due to the flexibility and good potentialities of this approach [48,49,50]. Bragato et al. and Calmanti et al. respectively reported the use of molybdate and tungstate-based ILs as effective catalysts for the cycloaddition of CO_2_ to epoxides [51,52]. Other authors have also reported encouraging results concerning the use of tungstate-based ILs for the catalysis reaction of CO_2_ with substrates like diamines, propargylic amines, aminophenols, aminothiobenzenes, and cyanoanilines for the preparation of corresponding cyclocarbonated compounds [53,54,55,56,57] or for the conversion of cyclic carbonates into linear ones [58]. In addition to this, tungstate salts have shown interesting catalytic performances in the catalysis of oxidative reactions like the preparations of carboxylic acids and aldehydes as well as sulfones and epoxides [59,60]. Concerning epoxidations, apart from classic tungstate salts, the use of tungstate ILs has also been investigated, producing positive outcomes [53,56]. Nevertheless, to the best of our knowledge, no papers in the literature have yet considered tungstate-based DILs. In the present study, we report the synthesis and characterization of a panel of bromide and tungstate-based DILs featuring imidazolium charged heads and rigid aromatic spacers. Imidazolium systems have been chosen for this study due to their great popularity, thus making possible a more direct comparison with structurally related DILs. Furthermore, the possibility of C2-H hydrogens forming hydrogen bonds with the anions considered in this work as well as with other molecules makes imidazolium DILs interesting both from the point of view of structural investigation and catalytic activity assessment [61]. The structures of the double-charged cations considered are reported in Figure 1. The effect of different spacers on the thermal properties of the proposed compounds has been assessed and a computational study of the relationship between the system conformations and the type of linker moiety has been carried out. An analysis of possible conformation of the minimal ion pairs with a comparison between bromide and tungstate DILs is reported in the computational part. Furthermore, we report some results of semiempirical molecular dynamics studies of these systems. Considering the attention given in the literature to the use of tungstate systems for CO_2_ activation reactions, we performed a proof-of-concept study of the applicability of the synthesised DILs in the catalysis of CO_2_’s addition to epoxides. Finally, the interactions occurring between tungstate anions and carbon dioxide in DILs–CO_2_ clusters were further explored by computational means.

## 2. Results and Discussion

### 2.1. Synthesis

A panel of six DILs featuring a xylyl semirigid spacer with bromide and tungstate anions was considered in this study (Figure 1). The prepared compounds have been characterised through ^1^H-NMR, ^13^C-NMR (Figures S1–S12, Supplementary Materials), and ATR-FTIR (Figures S13–S18, Supplementary Materials).

**Scheme 1:**
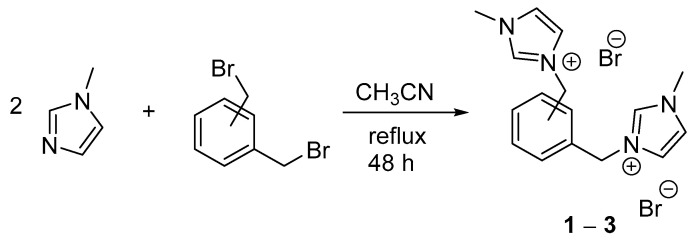
Synthesis of the bromide DILs (**1**–**3**).

**Scheme 2:**
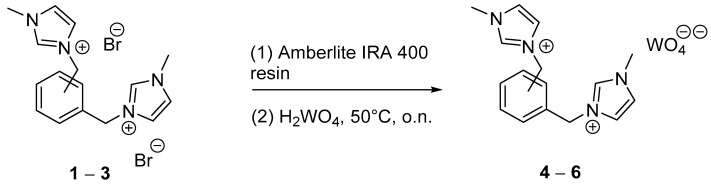
Synthesis of tungstate DILs (**4**–**6**).

The synthesis of bromide DILs **1**–**3** was performed following a literature procedure [62]. *N*-methyl imidazole and the selected di-halogenated xylenes were reacted in acetonitrile at solvent reflux temperature overnight, obtaining the desired compounds in an excellent yield (Scheme 1). Tungstate DILs **4**–**6** were prepared from the bromide DILs (Scheme 2). Differing from the synthesis of compounds **1**–**3**, the preparation of tungstate DILs **4**–**6** presented few difficulties. The first attempted approach relied on the metathesis reaction with Na_2_WO_4_, but it proved unsuccessful. The specific solubility properties of Na_2_WO_4_, of bromide DILs, and of the byproducts resulting from the ion metathesis made it impossible to find a suitable solvent for the ion exchange. A second attempt was made using Ag_2_WO_4_, prepared right before use, utilising the extremely low K_ps_ of the AgBr salt to push the equilibrium in favour of the desired products. This method proved to be only partially successful due to the presence of silver impurities in the obtained tungstate DILs, which we found impossible to remove. Eventually, the use of a procedure based on an ion exchange resin proved fully successful. Compounds **1**–**3** were firstly converted into the corresponding hydroxides using the ion exchange column, and the desired tungstate DILs were quantitatively obtained by reacting the hydroxide intermediates with H_2_WO_4_. The complete substitution of the halide anions during the preparation of the hydroxide intermediate was verified by a silver assay, as described in Section 3.5. IR analysis can be used as an effective method of verifying the formation of the desired tungstate product. Upon comparing the IR spectra of the DILs with bromide anions (Figures S13–S15, Supplementary Materials) and the spectra of the tungstate DILs (Figures S16–S18, Supplementary Materials), an intense peak associated with the WO_4_^2−^ group (Figure S19, Supplementary Materials) appeared in the last three spectra in the region at about 800 cm^−1^ (consistent with what was reported by Calmanti et al.) [52]. On the other hand, the signals characteristic of H_2_WO_4_, i.e., the peak at 940 cm^−1^ and the broad peak centred at about 650 cm^−1^, are not visible in the spectra of tungstate DILs **4**–**6**, indicating the absence of unreacted tungstic acid in the prepared compounds (Figure S20, Supplementary Materials).

### 2.2. Thermal Characterization

The thermal stability of compounds **1**–**6** was firstly investigated by thermogravimetric analysis (Figures S21–S26, Supplementary Materials). From the obtained data (Table 1), a direct connection was evident between the anion type and the thermal stability of the DIL. Compounds **1**–**3**, featuring bromide anions, displayed a degradation temperature almost 100 °C higher than their tungstate counterparts **4**–**6**. At the same time, the degradation profile itself appeared greatly influenced by the anion type. Bromide DILs (**1**–**3**) were characterised by a single degradation peak, even if sometimes they showed shoulder peaks or minor degradation peaks at higher temperatures (Figures S21–S23, Supplementary Materials). On the other hand, tungstate DILs displayed much more complicate profiles, with multiple degradation steps overlapping in the region between 200 °C and 400 °C, indicating a strong effect of the tungstate anion on the thermal degradation mechanisms (Figures S24–S26, Supplementary Materials). The nature of the linker chain also proved to strongly affect the thermal stability properties of compounds **1**–**6** (Table 1). *o*-xylenes spacers were found to provide compounds with a lower thermal stability when compared with *m*- and *p*-xylenes linkers, with this effect being more noticeable in tungstate DILs. Finally, the type of spacer was found to deeply affect the degradation profile of the studied compounds. Concerning bromides DILs, compound **1** (featuring the *o*-xylene linker) displayed a single and steep degradation step, while DIL **2** (featuring the *m*-xylene linker) still exhibited a quite simple degradation profile, mainly degrading in a single step but with a well-pronounced shoulder observable on the left side of the main degradation peak in the *d*weight%/*d*T plot (Figures S21 and S22, Supplementary Materials). Finally, the *p*-xylene DIL (**3**) displayed an intermediate behaviour between **1** and **2** (Figure S23, Supplementary Materials). It can therefore be concluded that rigid xylyl linker chains exert a much stronger effect on the thermal degradation profiles of DILs compared to the effect reported in literature for alkyl linker chains [29]. Concerning tungstate DILs **4**–**6**, the influence of the spacer on the thermal degradation profile was found to be even more noticeable. Compound **6**, featuring a *p*-xylene spacer, was found to degrade in a single step (Figure S26, Supplementary Materials). On the other hand, compounds **4** and **5** displayed a very complex degradation profile (Figures S24 and S25, Supplementary Materials).

### 2.3. Computational Results—DFT Studies

**Figure 2:**
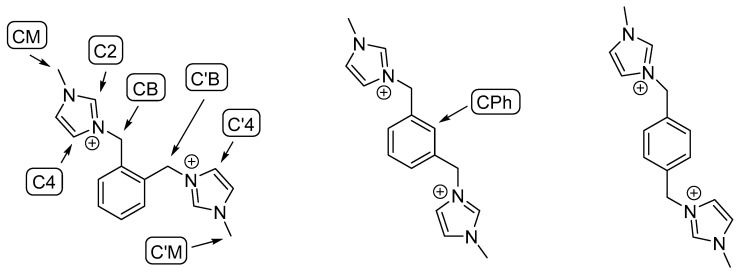
Dications with rigid spacers studied in this paper. From left to right: ortho, meta and para isomers. The labels indicate the C2, C4, CB[enzylic], CPh[enyl] positions used in this section.

**Figure 3:**
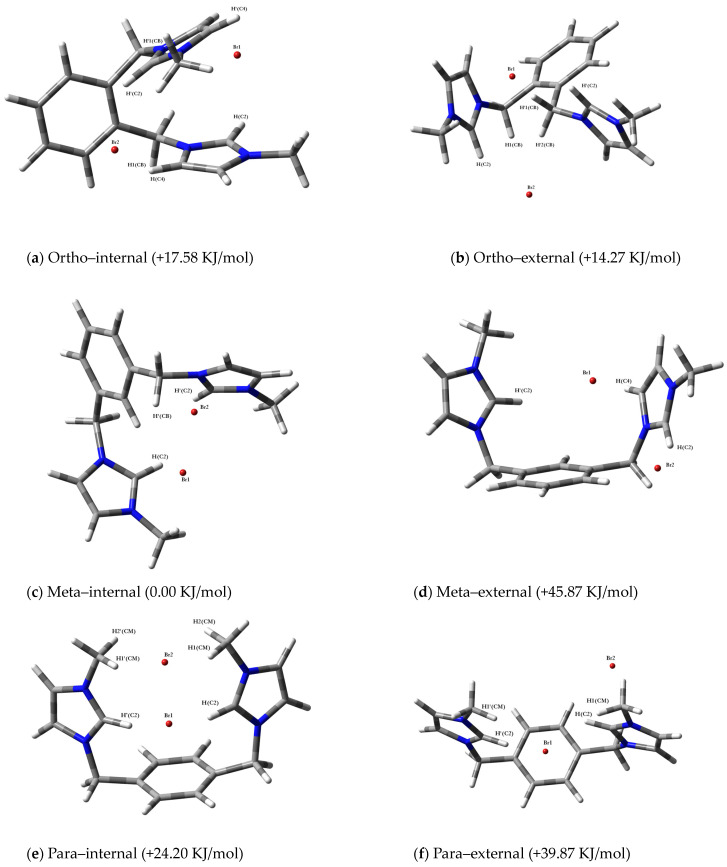
Optimised geometries of the minimal neutral clusters with bromide anions (**1**–**3**). Colour code for the atoms: hydrogen: white; carbon: grey; nitrogen: blue; bromine: red.

**Figure 4:**
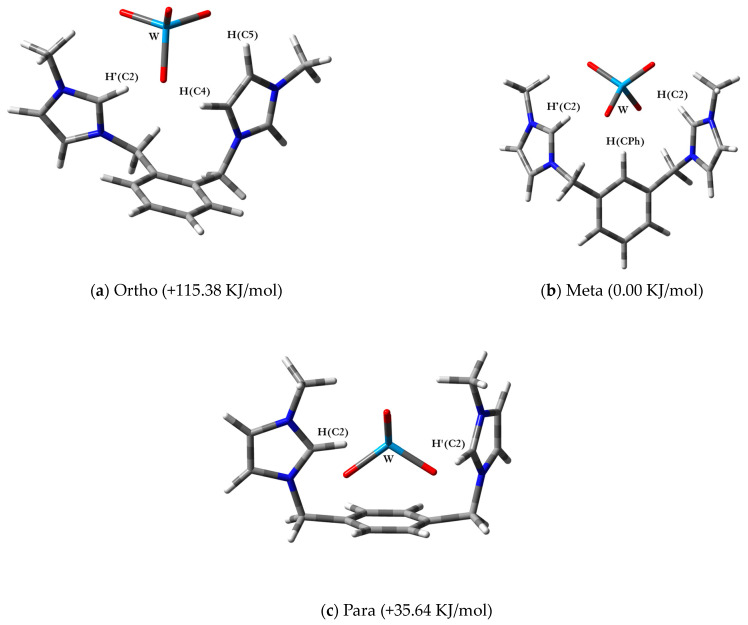
Optimised geometries of the minimal neutral clusters with tungstate anions (**4**–**6**). Colour code for the atoms: hydrogen: white; carbon: grey; nitrogen: blue; bromine: red; tungsten: cyan.

We chose to consider the minimum neutral clusters (dications reported in Figure 2 plus 2 Br^−^ or WO_4_^2−^) as a single supermolecular (or superionic) system given the fact that the strong electrostatic interactions involved lead to significant deformations of the geometry of the system. The optimised structures of compounds **1**–**6** are reported in Figure 3 (bromides **1**–**3**) and Figure 4 (tungstates **4**–**6**). Relative and absolute energies are reported in Table 2 (bromides **1**–**3**) and Table 3 (tungstates **4**–**6**). 

Systems with bromide anions are discussed first. For each isomer, we found two local minima with structural analogies, the first where anions are located between the two imidazolium rings (internal) and a second one (external) where at least one anion is outside the space between the rings.

The diverse geometry of the linker leads to differences in the spatial arrangement in terms of relative orientation and distances between the two imidazolium pendants (Figure 3). Concerning the ortho structures in **1**, in the internal conformer, the reduced distance between the two imidazolium rings forces the two anions to move apart. Conversely, in the external conformer, the different orientation of the rings allows a larger energetic stabilization. This is the only isomer where the external structure is more stable than the internal one. In the meta case **2,** there is enough space between the cationic headgroups to accommodate both bromide ions inside, leading to the most energetically stable conformation of the series and to the larger internal/external energetic gap. In the para isomer **3,** the two imidazolium pendants are diametrically opposite and the difference between the two conformers is therefore less well defined. In the symmetric internal structure, the anions can interact efficiently with both the imidazolium rings (Figure 3e). These structures are coherent with those reported by Verma et al. [64] where conformers very similar to the internal structures are reported for bromides and other single-charged anions. 

The scenario changes drastically with the tungstate DILs. While in the bromide case there are two independent anions, in the tungstate case, there is a single, spherical top and a sterically rigid dianion paired with a sterically flexible dication. In this case only, the analogous of the internal conformers is possible.

This different electrostatic and geometric scenario leads to remarkable differences in the geometrical arrangement of the dicationic structure depending on the type of anionic counterpart. From the electrostatic point of view, the C2-H moieties of the imidazolium rings seek energetic stabilization by pointing toward the negative charge centre. When the space between the two rings is small, like in the ortho tungstate **4** (Figure 4a), this is not possible and one of the rings must flip, pointing to its less polar backside (C4 and C5) towards the anion. On the other hand, with the same dicationic structure with two independent anions, as in the bromide case **1**, the conformation of the minimal neutral cluster adapts to a Yin–Yang-like structure where the two C2-H are anti-parallelly arranged, pointing to the two different bromides. In this case (both ortho structures in Figure 3a,b), there is a small energetic difference because the two structures are not so different. In the meta and para cases (**5** and **6**), there is enough space to host the WO_4_^2−^ anion with the rings aligned accordingly to the most energetically favourable arrangement. 

Looking at the energetic quantities, the pocket between the rings in the meta isomer is the most suitable for hosting both kinds of anionic counterparts, while the ortho is too small and the para too large. The internal meta/bromides (**2**, Figure 3c) and the meta/tungstate bromides (**5**, Figure 4b) are able to flip the benzene ring of the spacer in order to allow one hydrogen to point toward the anions. With respect to the other isomers where the benzene ring is parallelly oriented, this arrangement can stabilise the structure through the polarization of the aromatic system and by enhancing the dispersive ring–anion(s) interactions.

### 2.4. Computational Results—Molecular Dynamics Using the GFN2 Force Field

The dynamics of the bromide and tungstate DILs, the subject of this study (**4**–**6**), were assessed using the program xtb [65]. The parameters for the optimization were set to the standard procedure of the program; moreover, for the dynamics, the temperature was set to 298.15 K during a 2 ns simulation with a 2 fs step, and one structure was saved every 100 fs using the GFN2 force field [66]. The simulation was carried out in an NVT environment with a shake algorithm for all atoms.

These calculations have been performed to check the reliability of the force field for these kinds of systems and the stability of the above presented structures in a less static framework. 

**Figure 5:**
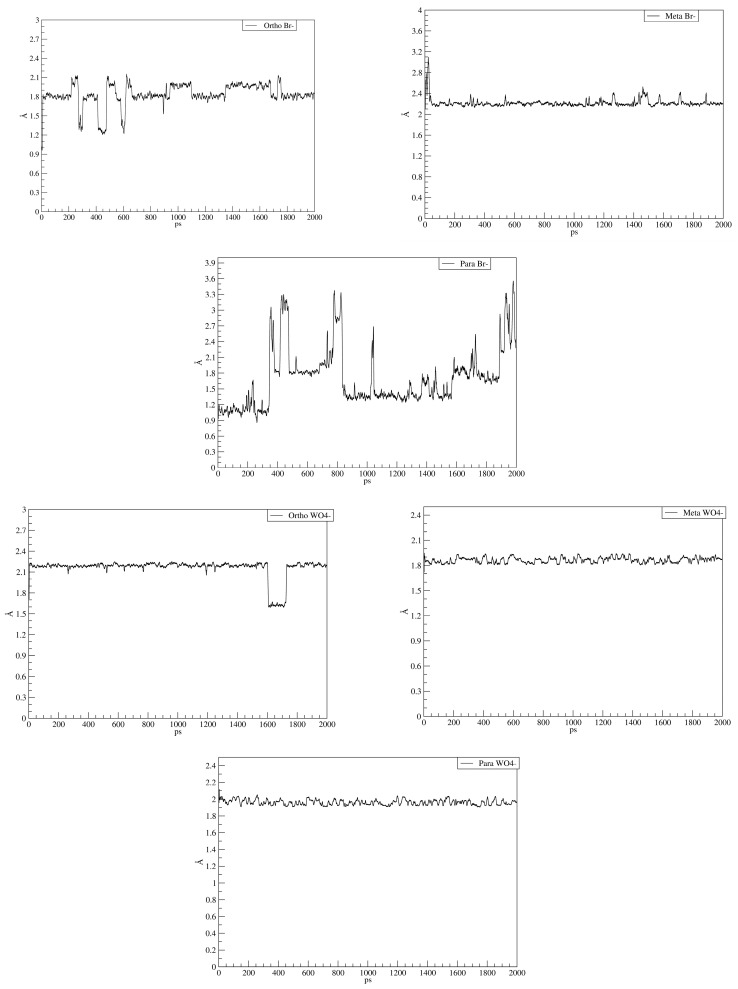
RMSD along the simulation time to the IL using the bromide anion **1**–**3** (top, ortho, meta and para conformations) and WO_4_^2−^ anion **4**–**6** (bottom, ortho, meta and para conformations).

The RMDS analysis, reported in Figure 5, shows a very different behaviour for systems with different anions. The bromide-based systems (**1**–**3**), which at the ab initio level showed two stable conformers, are more flexible and show larger oscillations, while the tungstate ones are more rigid. A similar behaviour can be concluded from the energetic profiles available in the Supplementary Materials (Figure S27). 

**Figure 6:**
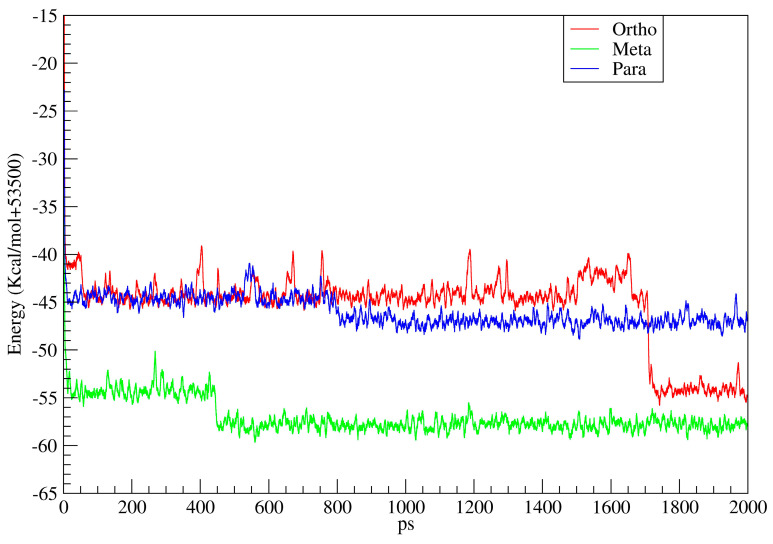
Energy variation along the simulation time to the IL using the tungstate anion (**4**–**6**) with the CO_2_ molecule system (conformations ortho, meta and para). Energies were shifted by 53,500 Kcal/mol in order to handle smaller numbers.

The reactivity of tungstate systems (**4**–**6**) with carbon dioxide was tested using the same level of theory. From the study, it emerged that tungstate DILs + CO_2_ systems are more energetically stable than their components for all the conformations. Interestingly, it was observed that a high variation in energy occurred in the ortho conformation cluster after 1.5 ns of simulation, and meta and para conformations displayed a variation in energy during the considered time of the analysis. However, this energy reduction was much lower when compared with the ortho cluster, and it was generated by a smaller change in conformation that created a more stable system (Figure 6). 

An RMSD analysis was performed to study the dynamic of the system and to gain a better understanding of the stability of the system. These data displayed characteristics like the ones observed for energy: the systems with the WO_4_^2−^ anions were more stable and presented a lower variation when compared with the Br^−^. 

This fact is due to different reasons:

1. The electrostatic effect of the double-charged WO_4_^2−^ anion is stronger. This has several effects on the geometry of the dications, the most evident being the stretching of the C2-H and C2′-H bonds (Table 3).
2. Differently from the WO_4_^2−^ systems, in the bromide case, there are two different anions that can be displaced independently. The dication/2Br^−^ systems therefore has a larger number of interionic degrees of freedom than the dication/WO_4_^2−^ ones. This was also demonstrated by the existence of the external conformations in the first case only.
3. The WO_4_^2−^ ion is bulkier than the bromide one. This lead to a greater sterical hindering and to a more rigid structure.

**Figure 7:**
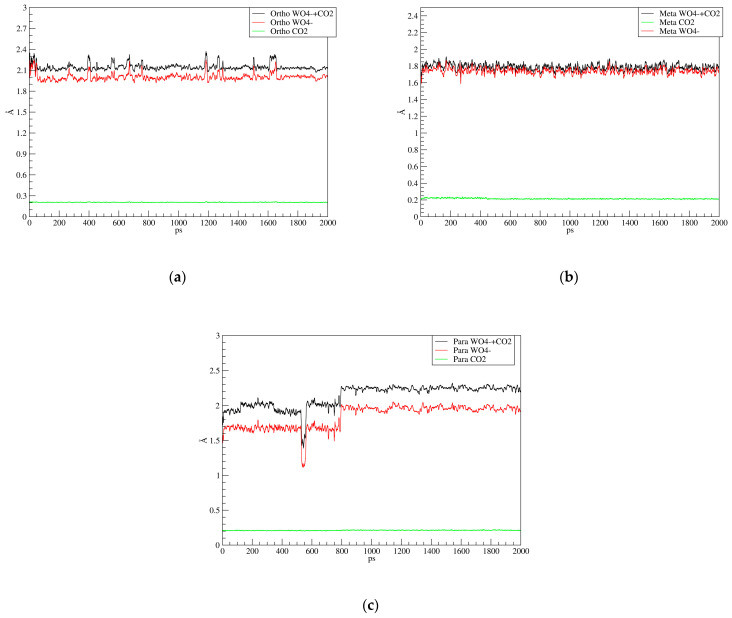
RMSD along the simulation time to the IL using the tungstate anion and CO_2_ molecule. (**a**) red: ortho DIL tungstate (**4**), black: ortho DIL tungstate (**4**) with CO_2_, green: ortho dication with CO_2_; (**b**) red: meta DIL tungstate (**5**), black: meta DIL tungstate (**5**) with CO_2_, green: meta dication with CO_2_; (**c**) red: para DIL tungstate (**6**), black: para DIL tungstate (**6**) with CO_2_, green: para dication with CO_2_.

As a further evidence of the structural rigidity and stability of tungstate DIL structures, the computational results concerning the interaction of these systems with CO_2_ showed only minimal structural variations with respect to the DIL before the coordination. Following the insertion of the carbon dioxide molecule, it interacted with the tungstate anion by forming an oxygen bridge, resulting in a carbonate-like structure, as depicted in Figure S31 (Supplementary Materials). The clusters where the CO_2_ interacted with the WO_4_^2−^ anion presented a stable RMSD for the meta system, the ortho system presented a small variation during the simulation, and the para system presented three different configurations (Figure 7). 

Concerning the Br^−^ system, the para conformation cluster presented a high variation after the first 400 ps and presented relative stability between 800 ps and 1.4 ns; then, the system returned to having high movement. This is due to the fact that the rigid internal conformation (Figure 3, bottom left) that presented several interactions between both the imidazolium rings (via H(C2), H′(C2), H1(CM), H2(CM), H1′(CM) and H2′(CM) polar hydrogens) and the anions was very rigid, and some of these bonds were disrupted when the geometry changed. This was only partially balanced by the simultaneous formation of some weaker electrostatic interactions. The ortho conformation presented an instability before 600 ps; however, it gained a more stable conformation during the remaining time, with some minor changes between two different states over time. The meta conformation provided the most stable simulation, presenting a lower variation and not manifesting any notable change in conformations during that time (Figure 5). is the latter was also the case when the internal/external energy gap was larger. 

Concerning the WO_4_^2−^ systems, they proved to be stable over time, with the ortho presenting a change in its conformation between 1.6 ns and 1.8 ns, (this conformation was observed only in this time range, and later it changed back to the initial configuration). This time, stability could be related to energy stability. Since the tungstate anion presented a high volume and at least four points that could create bonds, we observed more and stronger interactions between the cation and the anions which stabilised and made the structure more rigid and less mobile (Figure 6). The CO_2_ cluster was found to be stable for the ortho and the meta conformations, while the para system presented two variation points, creating three different relatively stable conformations (Figure 7). The first and the second conformations occurred before 800 ps, while after this time, the system evolved toward a third configuration which remained stable. These conformations were generated by a modification of the imidazolium rings’ positioning to accommodate the CO_2_ molecule. On the other hand, the molecule of CO_2_ was stable and did not change in conformation during any of the simulations.

**Figure 8:**
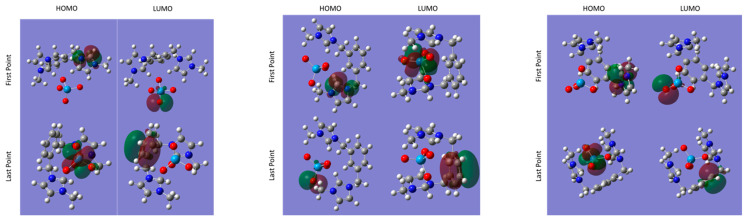
HOMO-LUMO analyses for the DILs with tungstate anion (**4**–**6**) in the ortho, meta, and para configurations (from left to right). Colour code for the atoms: hydrogen: white; carbon: grey; nitrogen: blue; bromine: red; tungsten: cyan.

Finally, to understand the changes that occur in these systems in the dynamic process, the differences between HOMO and LUMO in the first and the last geometrical structures of the MD runs are here reported. The structures of the DILs featuring bromide anions did not present significant differences (and thus are reported in the Supplementary Materials Figures S28–S30), while the tungstate ones are reported in Figure 8. Regarding the systems featuring the anion WO_4_^2−^, the charge distribution changesd during the simulation and created a new HOMO-LUMO configuration. At the starting point, the HOMO region was localised on the imidazolium ring on the region between the nitrogen atoms, while the LUMO region was found on the external oxygen atom of the WO_4_^2−^ anion, meaning that the oxygen did not interact with the cation. For the last point of the simulation, it has been observed that the HOMO region moved from the imidazolium ring to the tungstate anion in all the considered conformations, and that the LUMO region shifted to the central ring of the cation, indicating that the system changed its charge configuration as a result of the interaction with the anion. Moreover, it has been observed that the interaction of the oxygen atom created a stable configuration with both the imidazolium rings.

**Figure 9:**
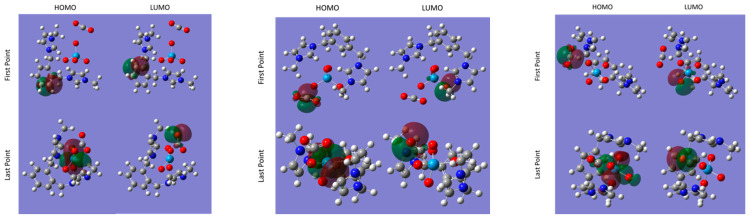
HOMO-LUMO analyses for the DILs with tungstate anion (**4**–**6**) with carbon dioxide in ortho, meta, and para configurations (from left to right). Colour code for the atoms: hydrogen: white; carbon: grey; nitrogen: blue; bromine: red; tungsten: cyan. Alternative versions of the last point structures without orbitals are reported in Supplementary Materials (Figures S28–S30).

The same analysis was performed on the systems with CO_2_ (Figure 9). Regarding these systems, at the beginning of the simulation, it was observed that not all the configurations presented the HOMO-LUMO regions in the same positions at the start point of the analysis. For the ortho configuration, the HOMO and the LUMO regions were concentrated on the central ring (the LUMO was located in the other half of the ring with respect to the HOMO), making it very reactive. The meta and para configurations instead presented the HOMO region on the CO_2_ molecule and the LUMO region on the external oxygen atom of the WO_4_^2−^ anion (the same configuration observed on the systems without CO_2_ molecules).

At the end of the simulation, the HOMO region was concentrated on the tungstate anion (a behaviour already observed when the CO_2_ molecule was not incorporated in the studied system), and the LUMO region was concentrated on the oxygen atom of the CO_2_ molecule, demonstrating the presence of an interaction between the molecule and the anion. This observation is consistent with literature data, and the good agreement with previous studies underlines the possibility of simulating this type of interaction [67]. The molecular dynamics using the GFN method demonstrated a good capability to simulate DIL systems both using a small anion (as the bromide) and a heavy anion (as the tungstate) and the interactions between them and other molecules. 

The energy analyses demonstrated that the increase in the anion size created a more stable system when combined with a dicationic IL and that the conformation of the molecule played a crucial role in the stabilization of the system. Concerning the bromide anion (**1**–**3**), the increase in the inter-ring space created a higher conformational energy and increased the movement of the system because of the increase in the mobility of the anion. However, for the tungstate anion (**4**–**6**), the increase in the inter-ring space created a lower conformational energy and reduced the movement due to the increase in the number of bonds and the better distribution of the charge throughout the system. When a carbon dioxide molecule was added to the tungstate system, it was observed that the system reorganised to favour the interactions between the anion of the DIL and the CO_2_ molecule. As observed in the literature for similar compounds, a partial charge transfer took place between the carbon dioxide and the tungstate [52,68]. This interaction can activate the carbon dioxide molecule toward various reactions, and it is therefore interesting for the design of catalysts for CO_2_ valorisation processes like the synthesis of cyclic carbonates, cyclic carbamates, and urea derivatives [52,55].

### 2.5. Proof-of-Concept Test of Compounds ***1***–***6*** as Catalysts for the of Cycloaddition of CO_2_ to Epoxides

**Scheme 3:**
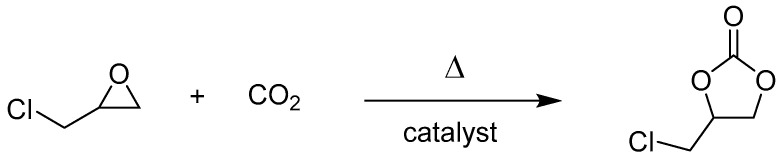
Schematic representation of the CCE under investigation.

Considering the good CO_2_ activation potential that emerged from the computational study on the proposed tungstate DILs, compounds **4**–**6** (and their parent bromide DILs **1**–**3**) were tested as catalysts for the cycloaddition of CO_2_ to epoxides (CCE reaction) (Scheme 3). Epichlorohydrin was chosen as the substrate due to its widespread use as a benchmark epoxide and the consequent possibility of comparing it with the results in the literature [51]. The reactions were performed with a CO_2_ pressure of 10 bar, a catalyst load of 1 mol%, a reaction time of 2 h, and temperatures of 80 °C and 100 °C.

**Figure 10:**
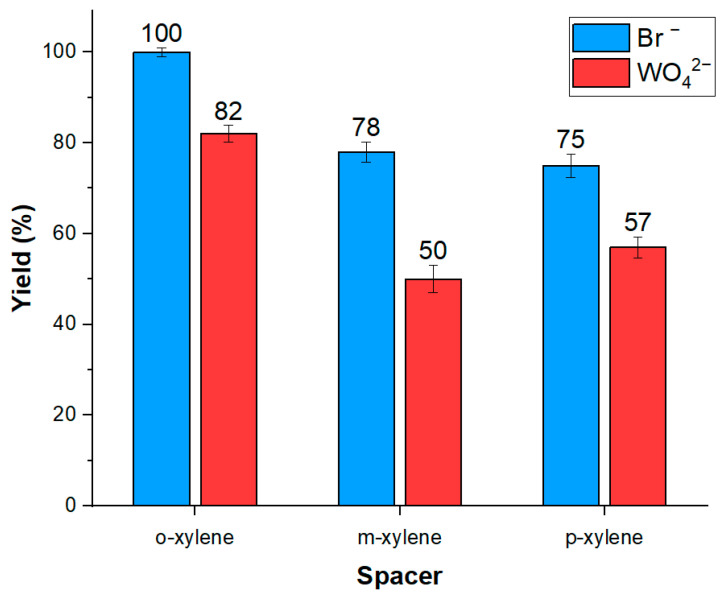
Results obtained for the CCE reaction performed on epichlorohydrin with bromide catalysts **1**–**3** (red) and tungstate catalysts **4**–**6** (blue). Reaction conditions: 1 mol% catalyst, 10 bar CO_2_, 100 °C, 2 h.

The obtained results show a clear relationship between the type of spacer and the catalytic activity of the DILs (Figure 10). In both the bromide (**1**–**3**) and the tungstate (**4**–**6**) DILs series, the compounds featuring an *o*-xylene (**1**, **4**) linker displayed the best performances in the CCE reaction, with **1** achieving a quantitative yield after 2 h at 100 °C and **4** reaching a yield of 82% in the same experimental conditions. All the reactions exhibited a complete selectivity, and no byproducts were observable by NMR spectroscopy. On the other hand, *m*-xylene (**2**, **5**), and *p*-xylene (**3**, **6**) DILs displayed a lower catalytic activity when compared to **1** and **4**, featuring an *o*-xylene spacer. Yields of 78% and 75% were observed, respectively, for bromides DILs **2** and **3**, while yields of 50% and 57% were observed, respectively, for tungstate DILs **5** and **6**. The significant difference in reactivity between the DILs featuring an *o*-xylene spacer and the other ones points out the great influence of the proximity of the imidazolium groups on the catalytic activity of the compound. Concerning the influence of the anion, bromide DILs proved more active than their tungstate counterparts, irrespectively of the type of linker moiety. These data, together with the computational results, provide some useful insights into the role of both imidazolium rigid dications and tungstate anions in catalysing the CCE reaction. From the calculations discussed in the previous section, an effective interaction between the tungstate anion and the carbon dioxide emerged. Nevertheless, if it is true that the energy data of DILs–CO_2_ clusters points out an activation effect exerted by the WO_4_^2−^ group on the CO_2_ molecule, it is evident that this activation alone is not sufficient to lead to a good catalytic activity. Moreover, it was noted that the dication conformations (meta and para) leading to the most stable dication–WO_4_^2−^ systems were also the ones leading to lower cyclic carbonate yields. The obtained results can be rationalised considering that the key role in the catalytic cycle is actually played by the activation of the epoxide. As reported in the literature, the activation of the epoxide ring is usually due to its interaction with a nucleophile, often assisted by the interaction with the oxygen of the epoxide ring with an electrophile (Scheme 3) [40]. Concerning the role of imidazolium systems in the electrophilic attack, the relatively acidic Im-H2 hydrogen (on the C2 of the imidazolium ring) is the atom more directly interacting with the epoxide. On the other hand, ionic liquid anions are well reported in the literature to effectively act as nucleophiles in the CCE reaction [36,40]. A final aspect to be considered concerns the capability of both the nucleophile and the electrophile to take part in the catalytic cycle; it is evident that hindered groups will less effectively activate the epoxide ring. With respect to their meta and para counterparts, ortho DILs manifest more accessible Im-H2 hydrogens according to our computational results (Figure 3 and Figure 4). This is particularly evident for the tungstate series, where **4** (Figure 4a), is the only tungstate DIL with a Im-H2 hydrogen pointing outward the ionic liquid structure. Furthermore, the higher mobility displayed by ortho structures **1** and **4** in the computational study (and thus their higher adaptability to their substrates) may represent another reason for their higher catalytic activity. The lower performances displayed by tungstate DILs **4**–**6** with respect to their bromide counterparts **1**–**3** can be rationalised considering both the nucleophilicity of the anion and the effect of WO_4_^2−^ on the structures of DILs **4**–**6**. In fact, the divalent anion WO_4_^2−^ confers a higher degree of rigidity to tungstate DILs (as discussed above in the computational section). In the second instance, the calculations display that the WO_4_^2−^ anion, bulkier than the Br^−^ anion, tends to occupy a good part of the concavity of the dication, yielding a packed structure where the accessibility to the groups involved in the catalysis of the CCE reaction was hindered (Figure 4). A second set of reactions was performed under the same experimental conditions but lowering the temperature to 80 °C in order to gain an insight into the effect of temperature on the catalytic activity of compounds **1**–**3**. Furthermore, the data collected from the reaction performed at 80 °C are directly comparable to data from the literature related to DILs featuring flexible linker chains, thus allowing us to evaluate the effect of rigid xylene spacers on the catalytic activity of bromide DILs. Unfortunately, compounds **1**–**3** were found to be only partially soluble in the reaction medium at this temperature, heavily affecting the results. The addition of epichlorohydrin carbonate (20 mol%) to the reaction mixture in order to promote the solubilisation of the catalysts only partially solved the problem, and the reacted mixture displayed considerable amounts of catalysts **1** and **3** still undissolved, while only **2** was found to be fully solubilised. This behaviour is in contrast with that reported in the literature regarding DILs with linear spacers, where the same amount of epichlorohydrin carbonate was sufficient to fully solubilise the least soluble catalyst reported in the study. It is evident that the presence of a rigid xylene-based spacer greatly affects the solubility of imidazolium DILs in epichlorohydrin, especially at a lower temperature Concerning the catalytic activity, the yield obtained using the DIL **2** as catalyst in the aforementioned experimental conditions (60%) appears consistent with that reported in the literature for bromide DILs featuring flexible propyl, butyl, pentyl, and hexyl linker chains in their structures (with yields of 57%, 62%, 67%, and 70%, respectively) [36].

## 3. Materials and Methods

All the employed reagents and solvents were used without further purifications. α,α′-dibromo-*o*-xylene, α,α′-dibromo-*m*-xylene, and α,α′-dibromo-*p*-xylene were purchased from TCI Chemicals—Europe N.V., Zwijndrecht, Belgium. *N*-methylimidazole, tungstic acid, and epichlorohydrin were obtained from Alpha Aesar (Thermo Fisher—Dreieich, Germany). Acetonitrile, ethyl acetate, methanol-d_4_ and sodium hydroxide were purchased from Merck Life Science S.r.l.—Darmstadt, Germany.

Fourier transform infrared spectroscopy (FTIR) spectra were registered using a Cary 600 Series FTIR spectrometer (Agilent Technologies, Santa Clara, CA, USA). Analyses were performed on a window of diamond with a length spanning from 5000 cm^−1^ to 550 cm^−1^, with 32 scans and resolution of 4 cm^−1^. ^1^H-NMR and ^13^C-NMR spectra were recorded with a Bruker 400 UltraShield operating at 400 MHz (^1^H-NMR) and 100 MHz (^13^C-NMR). The chemical shifts δ are referenced to either residual CD_3_OD (δ_H_ 3.31, δ_C_ 49.0) or D_2_O (δ_H_ 4.80), and J-values are given in Hz. The following abbreviations are used: s = singlet, d = doublet, dd = double doublet, m = multiplet. The intensity of the signal corresponding to the proton Im-H2 is heavily affected by the fast exchange of this proton in MeOD and D_2_O. 

Thermal gravimetric analyses were performed using a TA Instruments Q500 TGA (weighing precision ± 0.01%, sensitivity 0.1 µg, baseline dynamic drift < 50 µg). The Curie point of nickel standard was used to perform the temperature calibration, while weight standards of 1000, 500, and 100 mg were used for mass calibration. All the standards were supplied by TA Instruments Inc. Each sample (7–14 mg) was heated in a platinum crucible at 60 °C in N_2_ (100 mL/min) for 20 min. Then, the sample was heated from 40 to 700 °C (compounds **1**–**3**) or to 800 °C (compounds **4**–**6**) at the heating rate of 10 °C/min under nitrogen (50 mL/min). Mass change was recorded as a function of temperature and time. The thermal behaviour of the ionic liquids was analyzed using a differential scanning calorimeter DSC 250 (with a temperature accuracy of ± 0.05 °C, temperature precision of ± 0.008 °C, and enthalpy precision of ± 0.08%). The temperature calibration was performed considering the dependence of the heating rate on the onset temperature of the melting peak of indium. About 2–4 mg of sample was loaded into hermetic aluminium pans. Based on the results of TG analyses, DSC analyses were performed at 10 °C/min in N_2_ (50 mL/min) in a temperature range spanning from −90 to 150 °C. The sample underwent cycles of cooling and heating to the selected temperatures using scanning rates of 10 °C/min.

An EYELA PROCESS STATION PPV-4060 reactor (StepBio, Bologna, Italy), equipped with four autoclaves HIP-60 of stainless steel, was used for the cycloaddition of CO_2_ to epoxides. Each autoclave was equipped with a glass liner of 150 mL with a magnetic stirrer.

The geometrical structure of minimal neutral clusters was optimised using a Gaussian suite of programs with the B3LYP function, using the basis set with effective core potential LanL2DZ that allowed us to handle heavy atoms like tungsten and bromide at a reasonable computational cost [69,70]. All calculations were performed using the Gaussian16 package [71]. Some molecular dynamics studies with the xtb package, using the GFN2 tight-binding DFT [72], were performed on the six clusters studied above at the ab initio level. All the runs were 2000 ps in length. The starting structures corresponded to the optimised energies at the ab initio level. 

### 3.1. General Procedure for the Synthesis of 1,1′-(1,n-Phenylenebis(methylene))bis(3-methylimidazolium) Bromides (***1***–***3***)

A variation of the synthetic procedure reported by Magill et al. was followed for the preparation of compounds **1**–**3** [73]. In a flask containing a magnetic stirrer, 3.79 mmol (1.000 g) of the selected α,α′-dibromoxylene and 4 mL of CH_3_CN were added. To this mixture, a solution of 7.96 mmol (0.654 g) of *N*-methylimidazole in 6 mL of CH_3_CN was added dropwise, and the resulting mixture was heated to reflux overnight (80 °C). After cooling, the solution was concentrated, and the resulting solid was washed with 3 portions of 15 mL of EtOAc each then dried under reduced pressure. 

### 3.2. 1,1′-(1,2-Phenylenebis(methylene))bis(3-methylimidazolium) Bromide (***1***)

The title compound, prepared according to the general procedure using α,α′-dibromo-o-xylene, was recovered in a quantitative yield as a hygroscopic white solid.

^1^H-NMR (MeOD) *δ*: 9.06 (s, 2H, 2 × Im-*H*_2_), 7.64, 7.62 (2d, each 2H, *J*_vic_ = 2.0 Hz, 2 × Im-*H*_4_, 2 × Im-*H*_5_), 7.54, 7.41 (2m, each 2H, 4 × Ar-*H*), (m, 2H, Ar-*H*), 5.69 (s, 4H, 2 × PhC*H_2_*N), 3.97 (s, 6H, 2 × NC*H_3_*). ^13^C-NMR (MeOD) *δ* 138.1 (2 × Im-*C*_2_), 133.6 (2 × Ar-*C*), 131.5, 131.4 (4 × Ar-*C*H), 125.3, 123.8 (2 × Im-*C*_4_, 2 × Im-*C*_5_), 51.6 (2 × Ph*C*H_2_N), 36.9 (2 × N*C*H_3_).

FTIR (cm^−1^): 3136.3, 3073.8, 2938.8, 1645.0, 1560.4, 1453.6, 1427.9, 1362.9, 1334.9, 1156.7, 1091.6, 829.7, 754.8, 737.5, 660.0, 622.0.

The obtained NMR results (^1^H, ^13^C) were consistent with those reported in literature in DMSO [73].

### 3.3. 1,1′-(1,3-Phenylenebis(methylene))bis(3-methylimidazolium) Bromide (***2***)

The title compound, prepared according to the general procedure using α,α′-dibromo-m-xylene, was recovered in a quantitative yield as a hygroscopic white solid.

^1^H-NMR (MeOD) *δ*: 9.15 (s, 2H, 2 × Im-*H*_2_), 7.68 (m, 3H, 2 × Im-*H*_4_ or 2 × Im-*H*_5_ and Ar-*H*), 7.61(d, 2H, *J*_vic_ = 2.0 Hz, 2 × Im-*H*_4_ or 2 × Im-*H*_5_), 7.51 (s, 3H, Ar-*H*), 5.50 (s, 4H, 2 × PhC*H_2_*N), 3.96 (s, 6H, 2 × NC*H_3_*). ^13^C-NMR (MeOD) *δ* 138.3 (2 × Im-*C*_2_), 136.5 (2 × Ar-*C*), 131.4, 130.5, 130.2 (4 × Ar-*C*H), 125.3, 123.7 (2 × Im-*C*_4_, 2 × Im-*C*_5_), 53.5 (2 × Ph*C*H_2_N), 36.8 (2 × N*C*H_3_). 

FTIR (cm^−1^): 3138.2, 3063.4, 3024.2, 2952.5, 1651.4, 1577.3, 1557.3, 1452.4, 1427.4, 1361.6, 1337.9, 1170.1, 865.5, 787.1, 753.3, 737.1, 620.1.

The obtained NMR results (^1^H, ^13^C) were consistent with those reported in literature in DMSO [73].

### 3.4. 1,1′-(1,4-Phenylenebis(methylene))bis(3-methylimidazolium) Bromide (***3***)

The title compound, prepared according to the general procedure using α,α′-dibromo-p-xylene, was recovered in a quantitative yield as a hygroscopic white solid.

^1^H-NMR (MeOD) *δ*: 9.10 (s, 2H, 2 × Im-*H*_2_), 7.65, 7.61 (2d, each 2H, *J*_vic_ = 2.0 Hz, 2 × Im-*H*_4_, 2 × Im-*H*_5_), 7.55 (s, 4H, 4 × Ar-*H*), 5.48 (s, 4H, 2 × PhC*H_2_*N), 3.95 (s, 6H, 2 × NC*H_3_*). ^13^C-NMR (MeOD) *δ* 138.2 (2 × Im-*C*_2_), 136.4 (2 × Ar-*C*), 130.7 (4 × Ar-*C*H), 125.3, 123.7 (2 × Im-*C*_4_, 2 × Im-*C*_5_), 53.5 (2 × Ph*C*H_2_N), 36.8 (2 × N*C*H_3_).

FTIR (cm^−1^): 3109.0, 3037.9, 2936.7, 1645.7, 1572.0, 1557.6, 1361.0, 1323.8, 1161.5, 842.9, 762.3, 732.4, 618.3. 

The obtained NMR results (^1^H, ^13^C) were consistent with those reported in literature in DMSO [74].

### 3.5. General Procedure for the Synthesis of 1,1′-(1,N-phenylenebis(methylene))bis(3-methylimidazolium) Tungstates (***4***–***6***)

Compounds **4**–**6** were synthetised following a procedure previously reported by our research group [29]. Amberlite IRA 400 ion exchange resin (20 g) was firstly activated by stirring in NaOH 1M for 1 day at room temperature with a tilting stirrer. The resin was then packed in a column and washed several times with water until the excess of NaOH was eliminated. Prior to use, the column was conditioned with a solution of 75:25 (*v*/*v*) MeOH-H_2_O.

In a flask containing a magnetic stirrer, the selected bromide DIL (1.18 mmol, 0.505 g) was dissolved in 50 mL of a solution of 75:25 (*v*/*v*) MeOH-H_2_O and flowed through the column packed with the activated and conditioned IRA 400 resin. At the end of each passage through the column, the completion of the ion exchange was tested by AgNO_3_ essay to reveal the presence of residual halide anions. The test was performed by collecting a few drops of the solution after the column and diluting them up to 0.5 mL with deionised water. The obtained sample was neutralised by adding a drop of nitric acid (the neutralisation was checked with pH paper, and an excess of acid was found to not affect negatively the outcome of the essay). Finally, a drop of an aqueous solution of AgNO_3_ (5% *w*/*w*) was added to the sample. The formation of a white turbidity in the first seconds after the addition of the silver solution indicated the presence of residual halide anions. The DILs solution was recovered and flowed through the column until the test turned negative. A stoichiometric amount of H_2_WO_4_ (0.59 mmol, 0.147 g) was then added to the solution of DIL hydroxide and reacted for a night at 50 °C. The desired products were obtained in excellent yields after removal of the solvent under reduced pressure. The ion exchange resin was later regenerated with 250 mL NaOH 1M.

### 3.6. 1,1′-(1,2-Phenylenebis(methylene))bis(3-methylimidazolium) Tungstate (***4***)

The title compound was obtained as a hygroscopic pale yellow glassy solid with a yield of 95.1%.

^1^H-NMR (D_2_O) *δ*: 8.66 (s, 2H, 2 × Im-*H*_2_), 7.64 (m, 2H, 2 × Ar-*H*), 7.50, 7.40 (2d, each 2H, *J*_vic_ = 2.0 Hz, 2 × Im-*H*_4_, 2 × Im-*H*_5_), 7.48 (m, 2H, 2 × Ar-*H*), 5.54 (s, 4H, 2 × PhC*H_2_*N), 3.89 (s, 6H, 2 × NC*H_3_*). ^13^C-NMR (D_2_O) *δ*: 136.2, (2 × Im-*C*_2_), 131.5 (2 × Ar-*C*), 131.1, 130.6 (4 × Ar-*C*H), 123.8, 122.2 (2 × Im-C4, 2 × Im-C5), 50.0 (2 × Ph*C*H_2_N), 35.8 (2 × N*C*H_3_). 

FTIR (cm^−1^): 3139.3, 3066.0, 1650.0, 1570.7, 1452.9, 1159.5, 793.2.

### 3.7. 1,1′-(1,3-Phenylenebis(methylene))bis(3-methylimidazolium) Tungstate (***5***)

The title compound was obtained as a hygroscopic pale yellow glassy solid (92.9% yield).

^1^H-NMR (D_2_O) *δ*: 8.79 (s, 2H, 2 × Im-*H*_2_), 7.57 (m, 1H, Ar-*H*), 7.46 (m, 7H, 2 × Im-*H*_4_, 2 × Im-*H*_5_ and 3 × Ar-*H*), 5.44 (s, 4H, 2 × PhC*H_2_*N), 3.92 (s, 6H, 2 × NC*H_3_*). ^13^C-NMR (D_2_O) *δ*: 136.1 (2 × Im-*C*_2_), 134.6 (2 × Ar-*C*), 130.2, 129.2, 128.5 (4 × Ar-*C*H), 123.8, 122.2 (2 × Im-*C*_4_, 2 × Im-*C*_5_), 52.4 (2 × Ph*C*H_2_N), 35.8 (2 × N*C*H_3_). 

FTIR (cm^−1^): 3138.4, 3065.0, 2849.7, 1648.0, 1560.3, 1449.4, 1164.9, 792.9.

### 3.8. 1,1′-(1,4-Phenylenebis(methylene))bis(3-methylimidazolium) Tungstate (***6***)

The title compound was obtained as a hygroscopic white solid (94.9% yield).

^1^H-NMR (D_2_O) *δ*: 8.76 (s, 2H, 2 × Im-*H*_2_), 7.47–7.44 (m, 8H, 2 × Im-*H*_4_, 2 × Im-*H*_5_ and 4 × Ar-*H*), 5.42 (s, 4H, 2 × PhC*H_2_*N), 3.88 (s, 6H, 2 × NC*H_3_*). ^13^C-NMR (D_2_O) *δ*: 136.1 (2 × Im-*C*_2_), 134.5 (2 × Ar-*C*), 129.3 (4 × Ar-*C*H), 123.9, 122.3 (2 × Im-*C*_4_, 2 × Im-*C*_5_), 52.3 (2 × Ph*C*H_2_N), 35.8 (2 × N*C*H_3_).

FTIR (cm^−1^): 3138.0, 3091.4, 1650.3, 1557.7, 1454.1, 1430.0, 1154.5, 785.4, 723.6.

### 3.9. General Procedure for the DILs Catalyzed Cycloaddition of CO_2_ to Epichlorohydrin

Epichlorohydrin (86.1 mmol, 7.966 g) and the catalyst (1% mol) were stirred at the prefixed temperature and at 10 bar of CO_2_ pressure for 2 h (the details of the employed reaction conditions will be described and discussed in the results and discussion section). The yield was evaluated by ^1^H-NMR on the crude reaction mixture as an average of three runs. The uncertainty of the reported yields is always within ±3%. 

## 4. Conclusions

In this paper, the chemical space of dicationic ionic liquids (DILs) was further expanded by the synthesis, characterization, and analysis of a new class of compounds composed of different dications with aromatic spacers coupled with bromide and tungstate anions. 

The isomerism of the rigid spacers here considered (ortho, meta and para) leads to different potential conformations of the dications and different potential relative positions of the cationic and the anionic moieties. By computational means, it emerged that meta and para isomers presented a pocket between the two imidazolium rings that could host the bromide anions (or the double-charged tungstate anion), while in the case of the ortho isomer, this was not sterically possible. The molecular dynamics studies showed a good complexity and several potential organizations of these systems. Moreover, the computational study pointed out that the double-charged tungstate anion, when it could be well accommodated in the dication pocket, led to more stable structures. The lower thermal stability manifested by both the ortho DILs with respect to their meta and para counterparts is coherent with these computational results. 

The use of these systems as catalysts in the cyclic carbonate reaction provided strong evidence of the influence of the conformation and of the system mobility on their chemical properties. On one hand, the anion type proved decisive in defining the catalytic activity of the studied DILs, with bromide systems **1**–**3** clearly outperforming their tungstate counterparts **4**–**6**. On the other hand, the type of spacer proved equally important in both the series of DILs, with the ortho DILs showing a remarkably higher activity when compared with the corresponding meta and the para isomers. Based on the insights into the structural conformations of the proposed systems obtained in the computational study, it can be noted that the catalytic activity appeared intimately connected with the structural flexibility and adaptability of the catalyst. More specifically, the good catalytic performances of the ortho DILs were associated with lower cluster stabilities at the computational level. Furthermore, the computational data showed that in the ortho structures, the pocket formed by the imidazolium heads was too small to properly host the anions, allowing both the anions and the cationic heads to be more freely accessible by the epoxide substrate. The computational results finally indicated that the studied tungstate DILs could also have an activating effect on carbon dioxide, with the obtained data highlighting the existence of effective interactions between the WO_4_^2−^ anion and the molecule of CO_2_. Nevertheless, the experimental evidence obtained in this study shows that the catalytic role of the tested DILs is mainly determined by their interactions with the epoxide ring. The formation of the tungstate DILs—CO_2_ clusters still represents an interesting result, and the proposed systems may display good potential in applications in which the importance of the direct activation of carbon dioxide is preponderant.

## Figures and Tables

**Table 1:** The *T*_start 5%_, *T*_onset_, and *T*_peak_ of compounds **1**–**6**. *T*_start 5%_ is defined as the temperature at which the weight loss of the sample is equal to 5% of the initial mass; *T*_onset_ is defined as the temperature at the onset of the weight (%) vs. temperature curve; and *T*_peak_ is defined as the temperature of the highest peak in the *d*weight%/*d*T plot.

Compound	1	2	3	4	5	6
*T*_peak_ (°C)	334.2	361.5	355.2	214.8	251.2	260.3
*T*_onset_ (°C)	310.7	296.0	328.7	205.3	233.2	248.1
*T*_start 5%_ (°C)	281.2	284.5	299.7	217.2	240.2	253.1

**Table 2:** Energetic and selected geometrical quantities related to minimal neutral clusters with bromide anions. The carbon atoms labels are defined in Figure 2. Distances are reported in Å. ∆E + ∆ZPE are relative to the most stable isomer (meta) in the most stable conformation (internal). ∆∆E + ∆∆ZPE are the differences between the two conformations of the same isomer. The sum of hydrogen and bromide van der Waals radii fluctuate in the range 2.97–3.15 Å according to different compilations proposed in literature [63].

Isomer	Ortho		Meta		Para	
Conformer	Internal	External	Internal	External	Internal	External
Energies						
Abs. Energies (Ha)	−5988.938627	−5988.939888	−5988.945324	−5988.927851	−5988.936105	−5988.930137
∆E + ∆ZPE (KJ/mol)	+17.58	+14.27	0.00	+45.87	+24.20	+39.87
∆∆E + ∆∆ZPE (KJ/mol)		−3.31		+45.87		+15.67
distances/Å						
H(C2)-Br1	2.49	-	2.66	-	2.48	2.97
H(C2)-Br2	-	2.50	3.38	2.25	-	3.01
H′(C2)-Br1	-	2.41	2.42	2.89	2.48	2.36
H′(C2)-Br2	2.43	-	3.95	-	-	-
H(C4)-Br1	2.80	-	-	2.53	-	-
H′(C4)-Br2	2.63	-	-	-	-	-
H1(CB)-Br2	2.65	2.64	2.79	-	-	-
H1′(CB)-Br1	2.89	2.48	-	-	-	-
H2′(CB)-Br2	-	2.70	-	-	-	-
H1(CM)-Br1	-	-	-	-	2.98	2.63
H2(CM)-Br2	-	-	-	-	2.84	-
H1′(CM)-Br1	-	-	-	-	2.98	2.86
H2′(CM)-Br2	-	-	-	-	2.85	-

**Table 3:** Top: energetic and selected geometrical quantities related to neutral minimal neutral clusters with tungstate anions. The carbon atom labels are defined in Figure 2. Distances are reported in Å.

Isomer	Ortho	Meta	Para
Abs. energies (Ha)	−1209.542873	−1209.586818	−1209.573244
∆E + ∆ZPE (KJ/mol)	+115.38	0.00	+35.64
C2-H	1.08	1.16	1.09
C2′-H	1.10	1.08	1.13
H(C2)-W		2.78	2.82
H′(C2)-W	2.67	2.80	2.80
H(C4)-W	3.13		
H(C5)-W	3.21		
H(CPh)-W		2.91	
W-O (average)		1.78	1.79

## Data Availability

Data are contained within the article or the Supplementary Materials.

## Supplementary Materials

The following supporting information can be downloaded at: https://www.mdpi.com/article/10.3390/molecules29092131/s1, Figure S1. ^1^H-NMR spectrum of 1,1′-(1,2-phenylenebis(methylene))bis(3-methylimidazolium) bromide (**1**). Figure S2. ^13^C-NMR spectrum of 1,1′-(1,2-phenylenebis(methylene))bis(3-methylimidazolium) bromide (**1**). Figure S3. ^1^H-NMR spectrum of 1,1′-(1,3-phenylenebis(methylene))bis(3-methylimidazolium) bromide (**2**). Figure S4. ^13^C-NMR spectrum of 1,1′-(1,3-phenylenebis(methylene))bis(3-methylimidazolium) bromide (**2**). Figure S5. ^1^H-NMR spectrum of 1,1′-(1,4-phenylenebis(methylene))bis(3-methylimidazolium) bromide (**3**). Figure S6. ^13^C-NMR spectrum of 1,1′-(1,4-phenylenebis(methylene))bis(3-methylimidazolium) bromide (**3**). Figure S7. ^1^H-NMR spectrum of 1,1′-(1,2-phenylenebis(methylene))bis(3-methylimidazolium) tungstate (**4**). Figure S8. ^13^C-NMR spectrum of 1,1′-(1,2-phenylenebis(methylene))bis(3-methylimidazolium) tungstate (**4**). Figure S9. ^1^H-NMR spectrum of 1,1′-(1,3-phenylenebis(methylene))bis(3-methylimidazolium) tungstate (**5**). Figure S10. ^13^C-NMR spectrum of 1,1′-(1,3-phenylenebis(methylene))bis(3-methylimidazolium) tungstate (**5**). Figure S11. ^1^H-NMR spectrum of 1,1′-(1,4-phenylenebis(methylene))bis(3-methylimidazolium) tungstate (**6**). Figure S12. ^13^C-NMR spectrum of 1,1′-(1,4-phenylenebis(methylene))bis(3-methylimidazolium) tungstate (**6**). Figure S13. ATR-FTIR spectrum of 1,1′-(1,2-phenylenebis(methylene))bis(3-methylimidazolium) bromide (**1**). Figure S14. ATR-FTIR spectrum of 1,1′-(1,3-phenylenebis(methylene))bis(3-methylimidazolium) bromide (**2**). Figure S15. ATR-FTIR spectrum of 1,1′-(1,4-phenylenebis(methylene))bis(3-methylimidazolium) bromide (**3**). Figure S16. ATR-FTIR spectrum of 1,1′-(1,2-phenylenebis(methylene))bis(3-methylimidazolium) tungstate (**4**). Figure S17. ATR-FTIR spectrum of 1,1′-(1,3-phenylenebis(methylene))bis(3-methylimidazolium) tungstate (**5**). Figure S18. ATR-FTIR spectrum of 1,1′-(1,4-phenylenebis(methylene))bis(3-methylimidazolium) tungstate (**6**). Figure S19. ATR-FTIR spectrum of sodium tungstate. Figure S20. ATR-FTIR spectrum of tungstic acid. Figure S21. Thermal gravimetric analysis and derivative of 1,1′-(1,2-phenylenebis(methylene))bis(3-methylimidazolium) bromide (**1**). Figure S22. Thermal gravimetric analysis and derivative of 1,1′-(1,3-phenylenebis(methylene))bis(3-methylimidazolium) bromide (**2**). Figure S23. Thermal gravimetric analysis and derivative of 1,1′-(1,4-phenylenebis(methylene))bis(3-methylimidazolium) bromide (**3**). Figure S24. Thermal gravimetric analysis and derivative of 1,1′-(1,2-phenylenebis(methylene))bis(3-methylimidazolium) tungstate (**4**). Figure S25. Thermal gravimetric analysis and derivative of 1,1′-(1,3-phenylenebis(methylene))bis(3-methylimidazolium) tungstate (**5**). Figure S26. Thermal gravimetric analysis and derivative of 1,1′-(1,4-phenylenebis(methylene))bis(3-methylimidazolium) tungstate (**6**). Figure S27. Energy variation along the simulation time to the IL using the Bromide anion (Conformations Ortho, Meta and Para). Figure S28. HOMO-LUMO analyses for the IL with bromide anion in the configuration ortho. Figure S29. HOMO-LUMO analyses for the IL with bromide anion in the configuration meta. Figure S30. HOMO-LUMO analyses for the IL with bromide anion in the configuration para. Figure S31. Structure of cluster with CO_2_. Ortho configuration. Figure S32. Structure of cluster with CO_2_. Meta configuration. Figure S33. Structure of cluster with CO_2_. Para configuration.
