# Supplementary material for: A Combined Experimental/Computational Study of Dicationic Ionic Liquids with Bromide and Tungstate Anions
Source: Molecules. 2024 May 3;29(9):2131. doi: 10.3390/molecules29092131 (PMC11326805; doi:10.3390/molecules29092131)
Supplement: Supplementary file 1 [file molecules-29-02131-s001.zip › molecules-2942456-supplementary.pdf]

A Combined Experimental/Computational Study of Dicationic Ionic Liquids with Bromide and Tungstate Anions

Guelber Cardoso Gomes [1,†], Claudio Ferdeghini [1,†], Luca Guglielmero [2,*], Felicia D'Andrea [1], Lorenzo Guazzelli [1], Andrea Mezzetta [1] and Christian Silvio Pomelli [1,*]

[1] Department of Pharmacy, University of Pisa, Via Bonanno 33, 56126 Pisa, Italy; guelbercardoso@gmail.com (G.C.G.); claudioferdeghini@gmail.com (C.F.); felicia.dandrea@unipi.it (F.D.); lorenzo.guazzelli@unipi.it (L.G.); andrea.mezzetta@unipi.it (A.M.)
[2] Classe di Scienze, Scuola Normale Superiore, Piazza dei Cavalieri 7, 56126 Pisa, Italy
* Correspondence: luca.guglielmero@sns.it (L.G.); christian.pomelli@unipi.it (C.S.P.)
† These authors contributed equally to this work.

Supporting Information

Table of Contents

^{1}H-NMR and ^{13}C-NMR spectra of compounds **1-6** pages S2-S7

ATR-FTIR spectra of compounds **1-6** pages S8-S11

TGA profiles of compounds **1-6** pages S12-S14

Energy profile for molecular dynamics page S15

HOMO and LUMO orbitals for **1-3** pages S15-S17

Structures of WO_4^{2-}+CO_2 clusters pages S18-S19

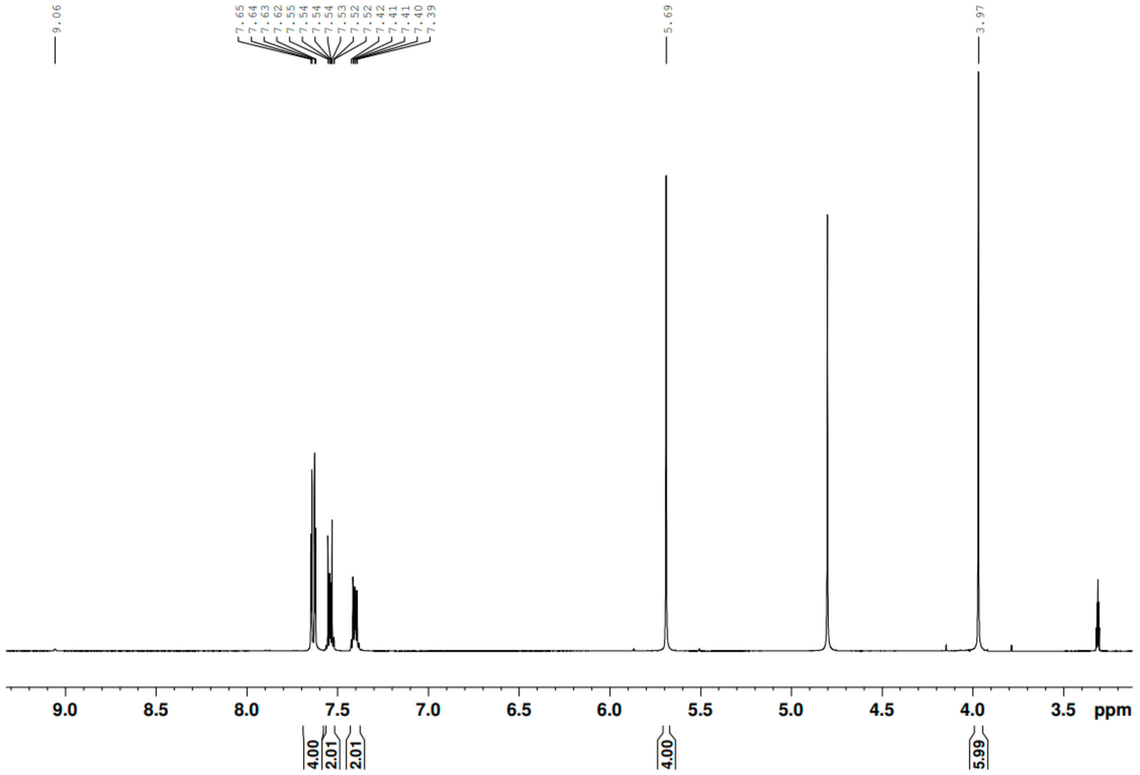

Figure S1. ^{1}H-NMR spectrum of 1,1'-(1,2-phenylenebis(methylene))bis(3-methylimidazolium) bromide (**1**).

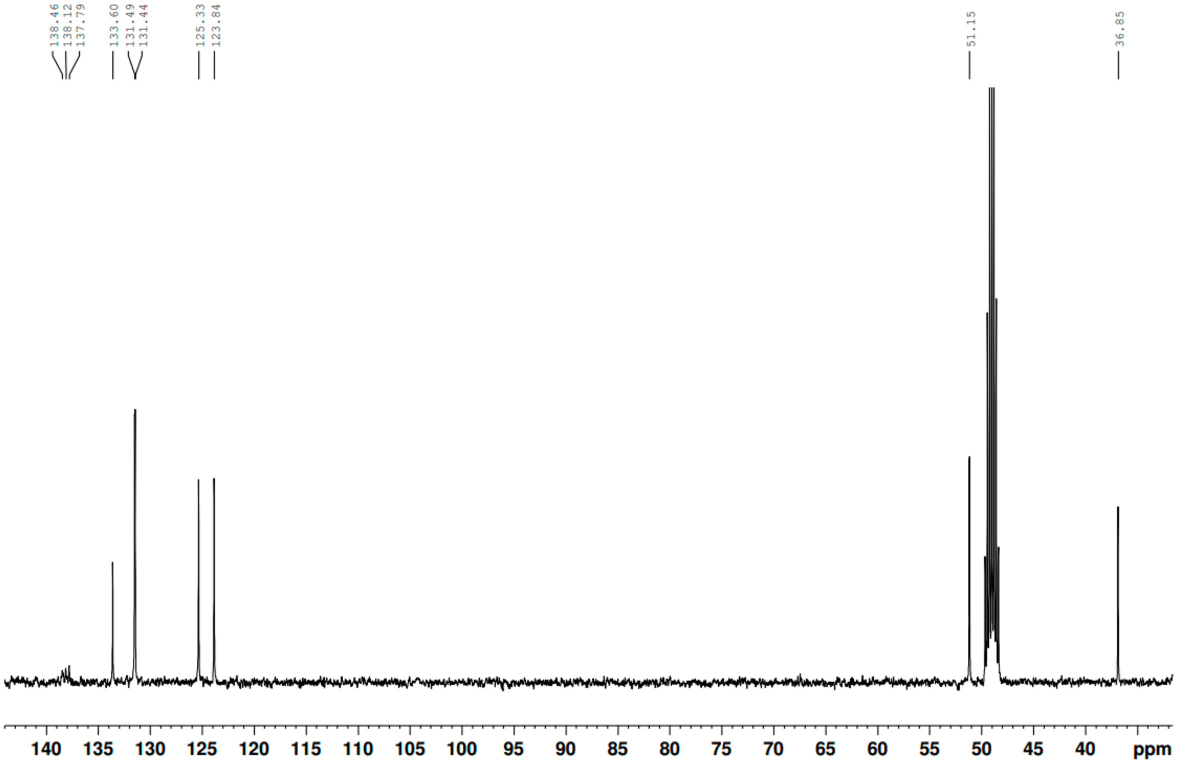

Figure S2. ^{13}C-NMR spectrum of 1,1'-(1,2-phenylenebis(methylene))bis(3-methylimidazolium) bromide (**1**).

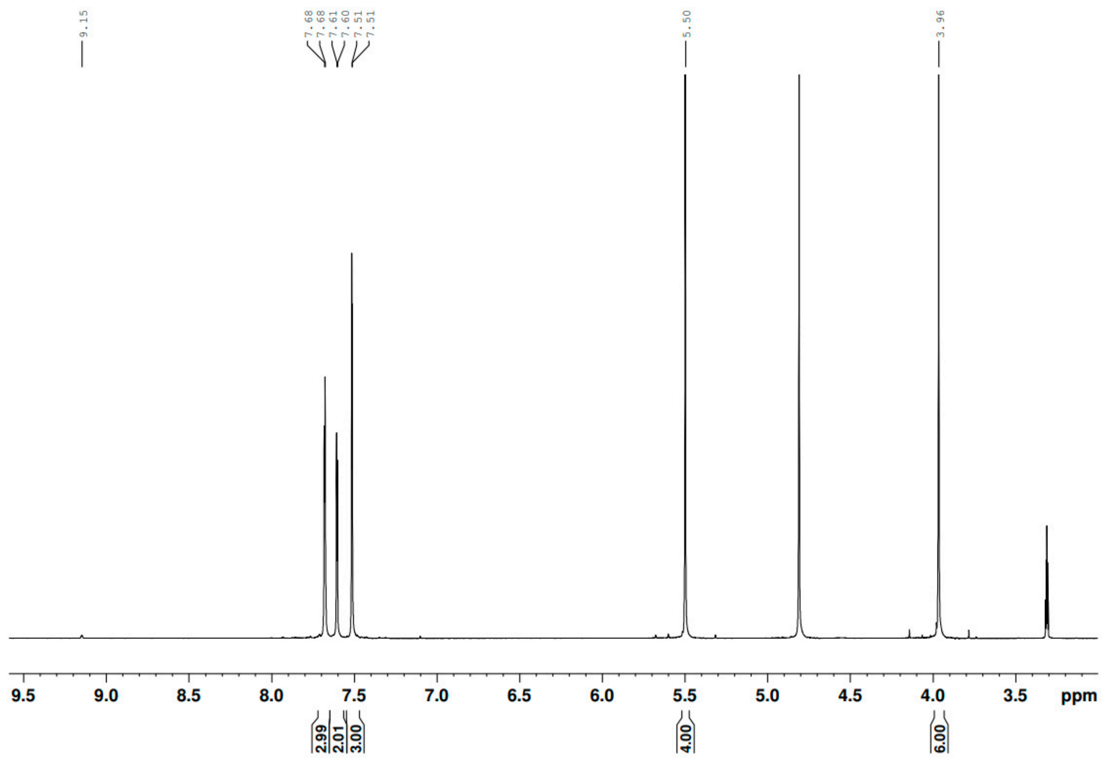

Figure S3. ^{1}H-NMR spectrum of 1,1'-(1,3-phenylenebis(methylene))bis(3-methylimidazolium) bromide (**2**).

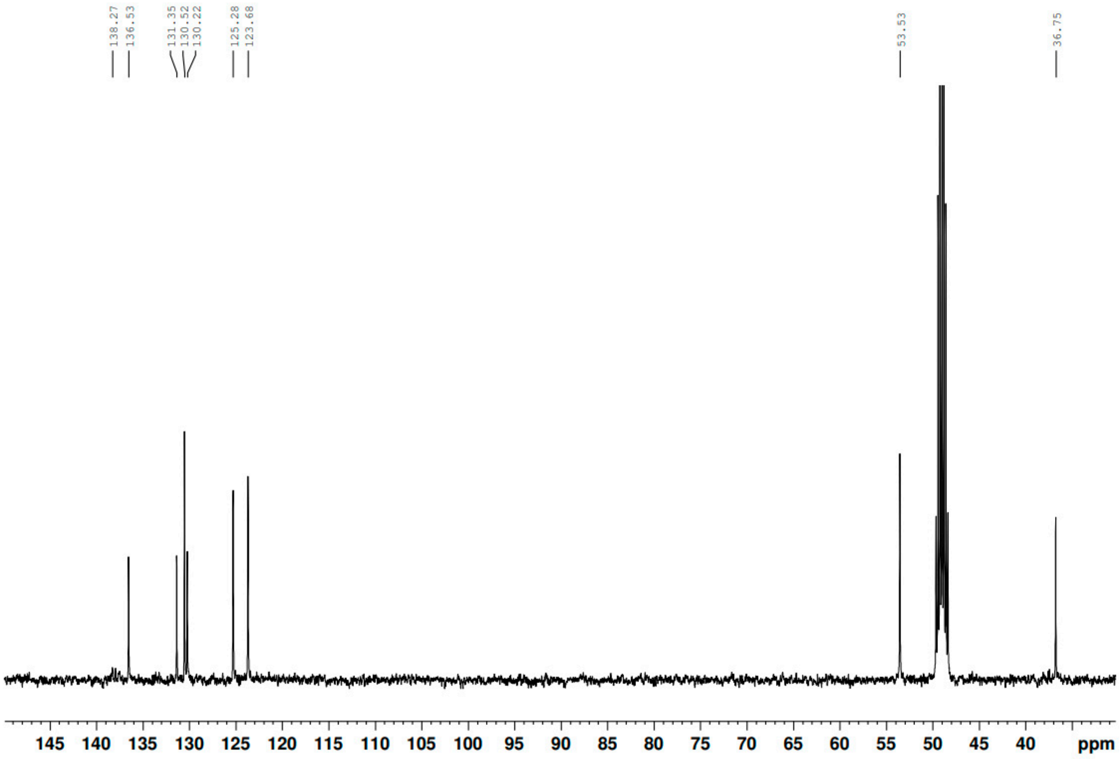

Figure S4. ^{13}C-NMR spectrum of 1,1'-(1,3-phenylenebis(methylene))bis(3-methylimidazolium) bromide (**2**).

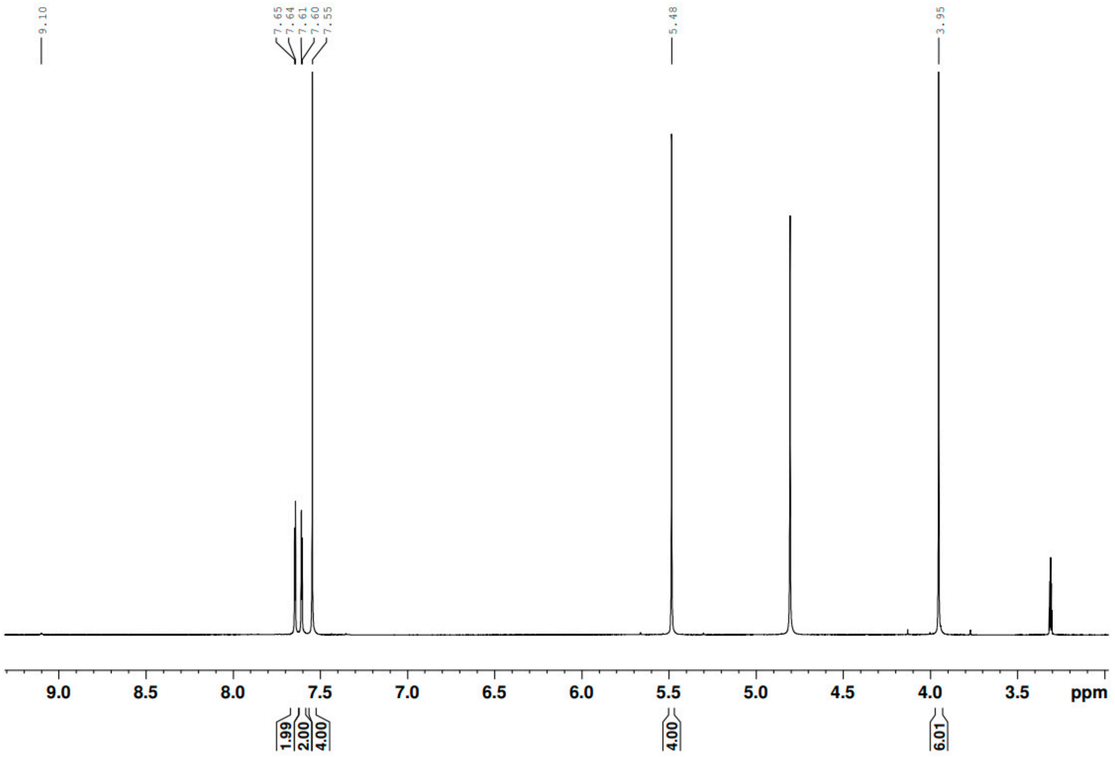

Figure S5. ^{1}H-NMR spectrum of 1,1'-(1,4-phenylenebis(methylene))bis(3-methylimidazolium) bromide (**3**).

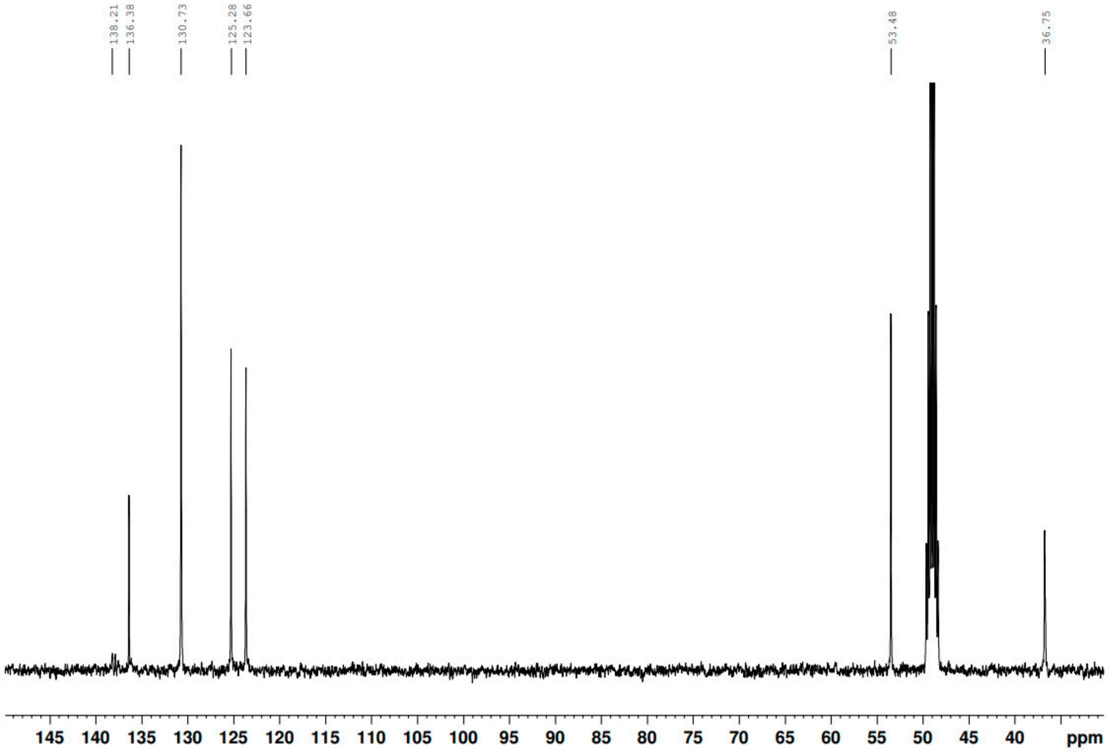

Figure S6. ^{13}C-NMR spectrum of 1,1'-(1,4-phenylenebis(methylene))bis(3-methylimidazolium) bromide (**3**).

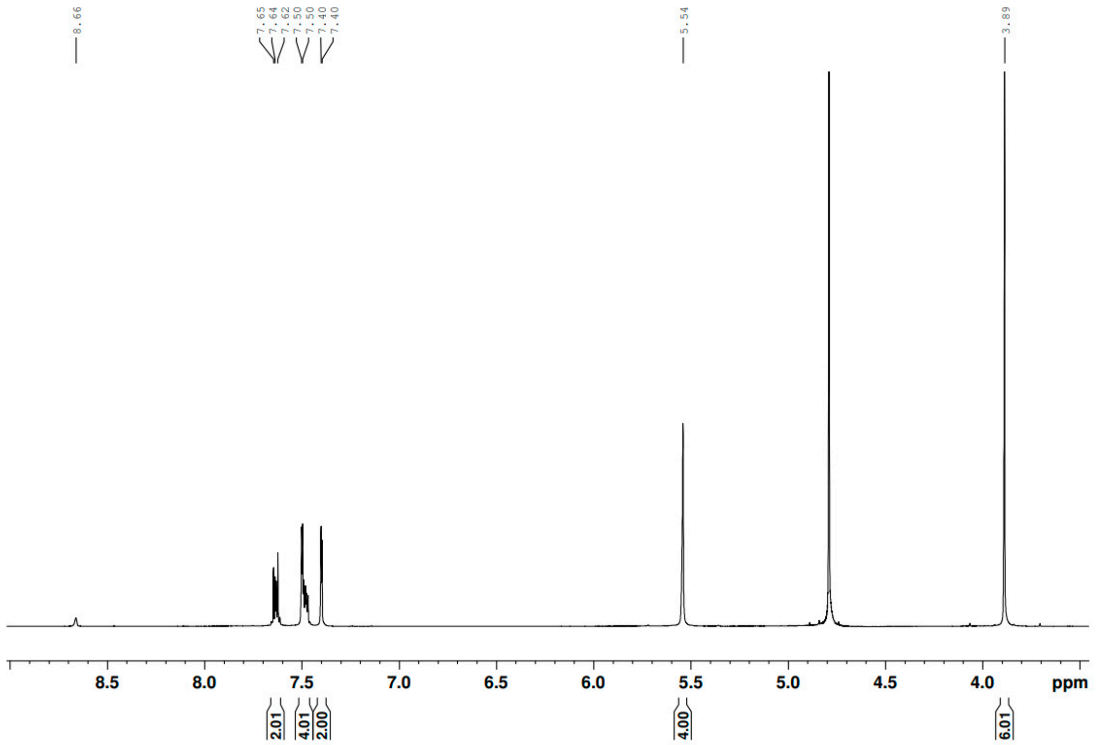

Figure S7. ^{1}H-NMR spectrum of 1,1'-(1,2-phenylenebis(methylene))bis(3-methylimidazolium) tungstate (**4**).

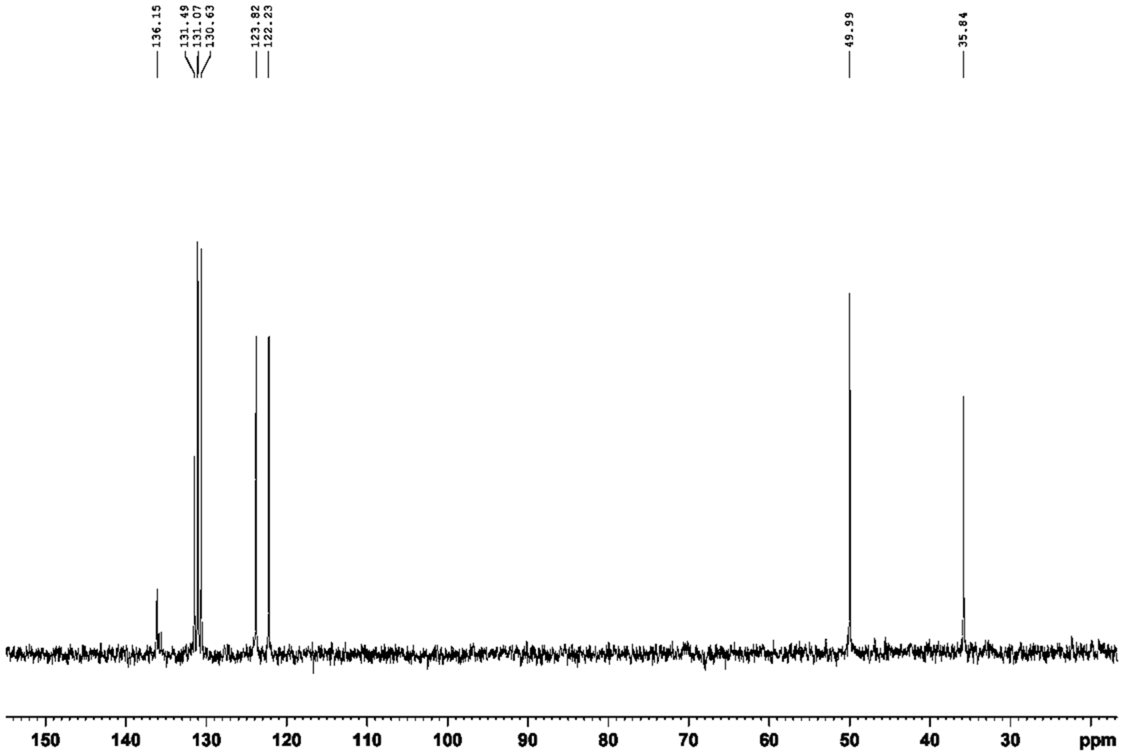

Figure S8. ^{13}C-NMR spectrum of 1,1'-(1,2-phenylenebis(methylene))bis(3-methylimidazolium) tungstate (**4**).

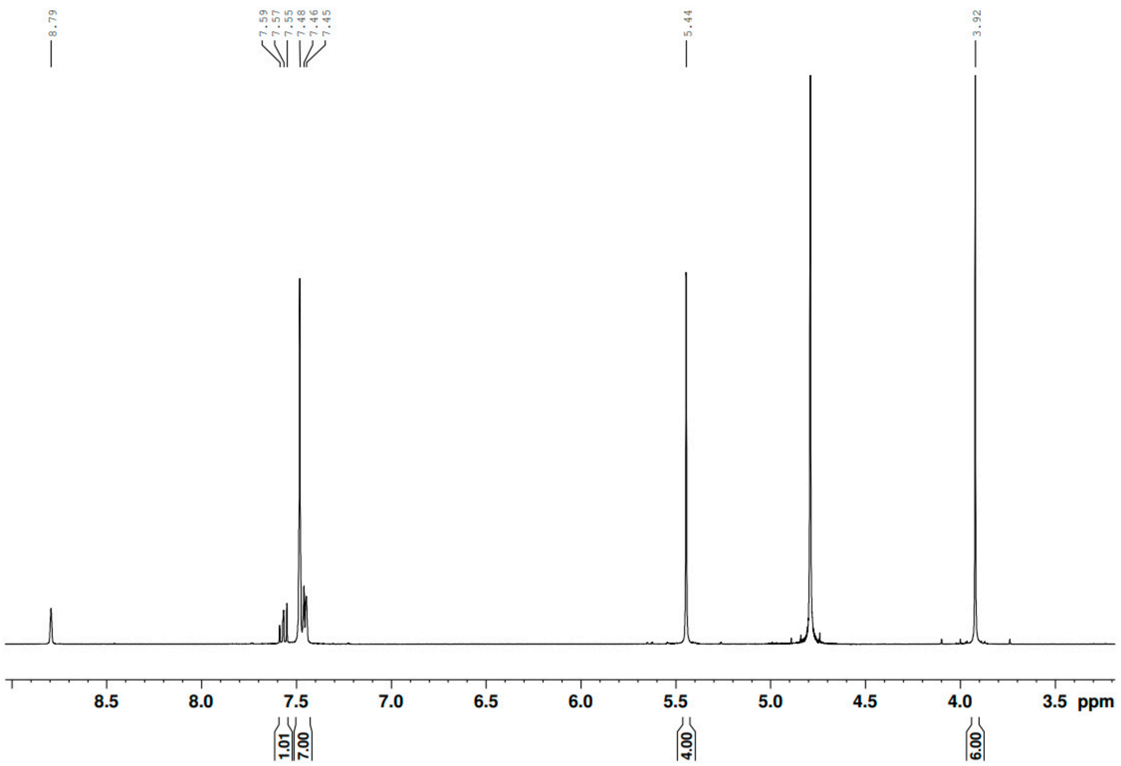

Figure S9. ^{1}H-NMR spectrum of 1,1'-(1,3-phenylenebis(methylene))bis(3-methylimidazolium) tungstate (**5**).

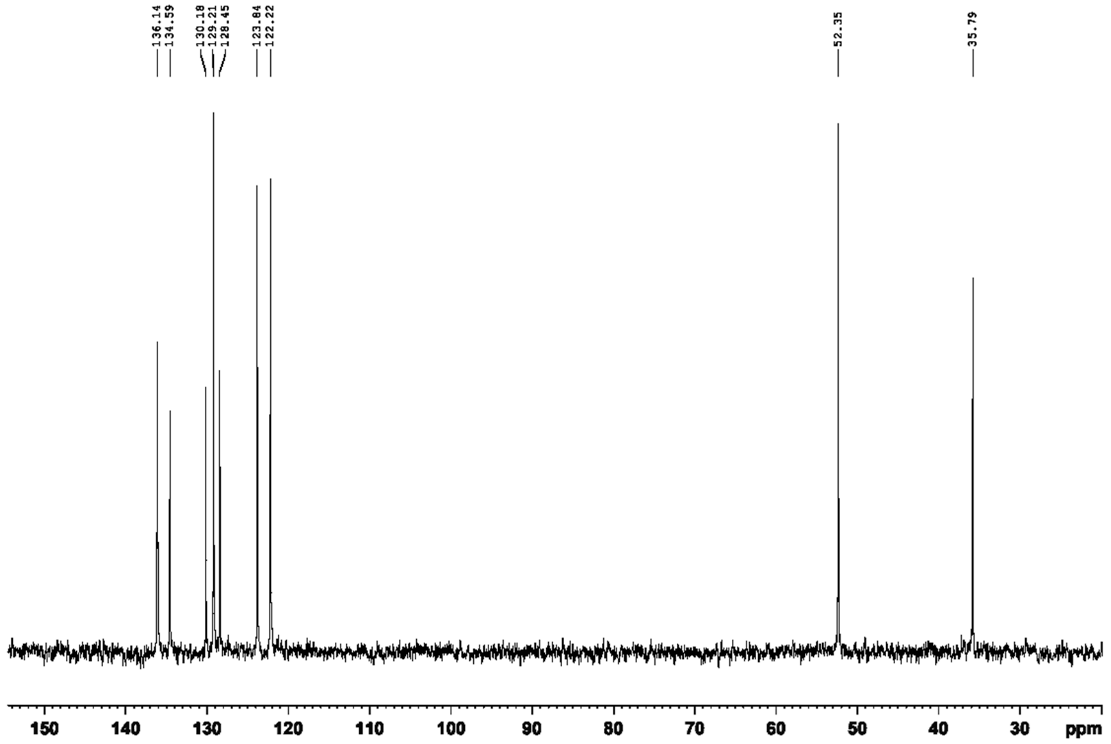

Figure S10. ^{13}C-NMR spectrum of 1,1'-(1,3-phenylenebis(methylene))bis(3-methylimidazolium) tungstate (**5**).

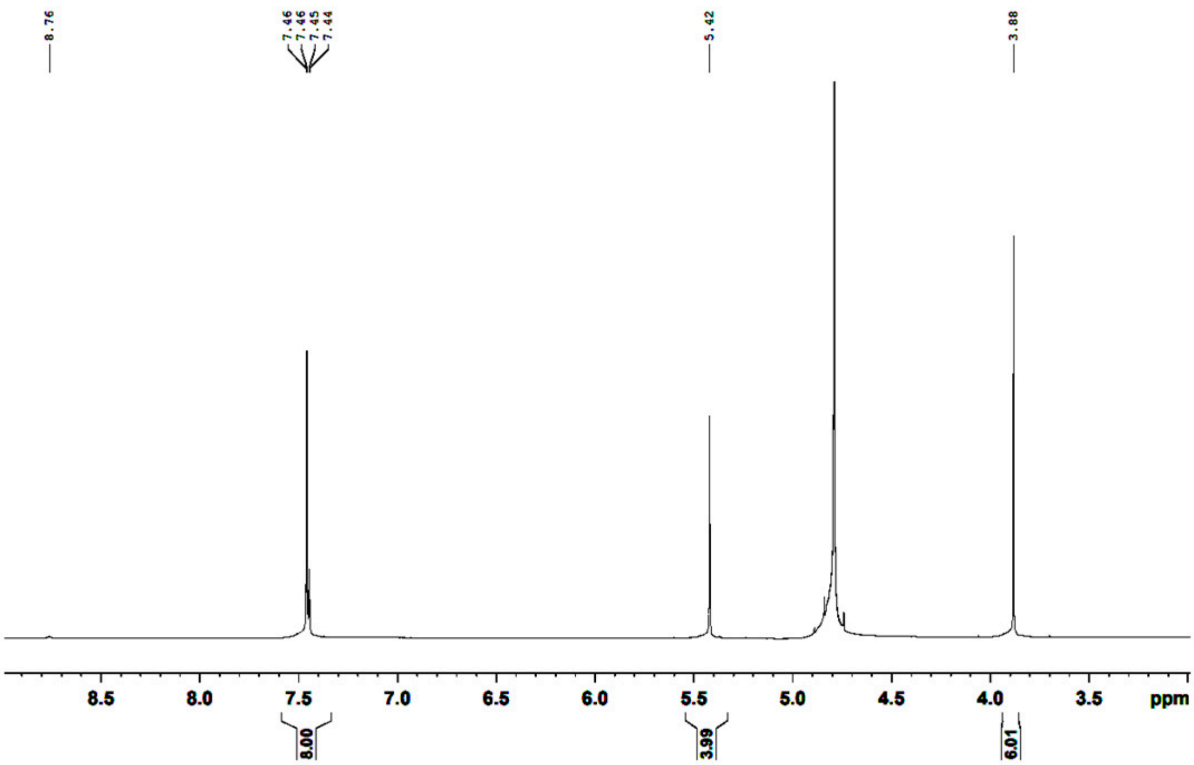

Figure S11. ^{1}H-NMR spectrum of 1,1'-(1,4-phenylenebis(methylene))bis(3-methylimidazolium) tungstate (**6**).

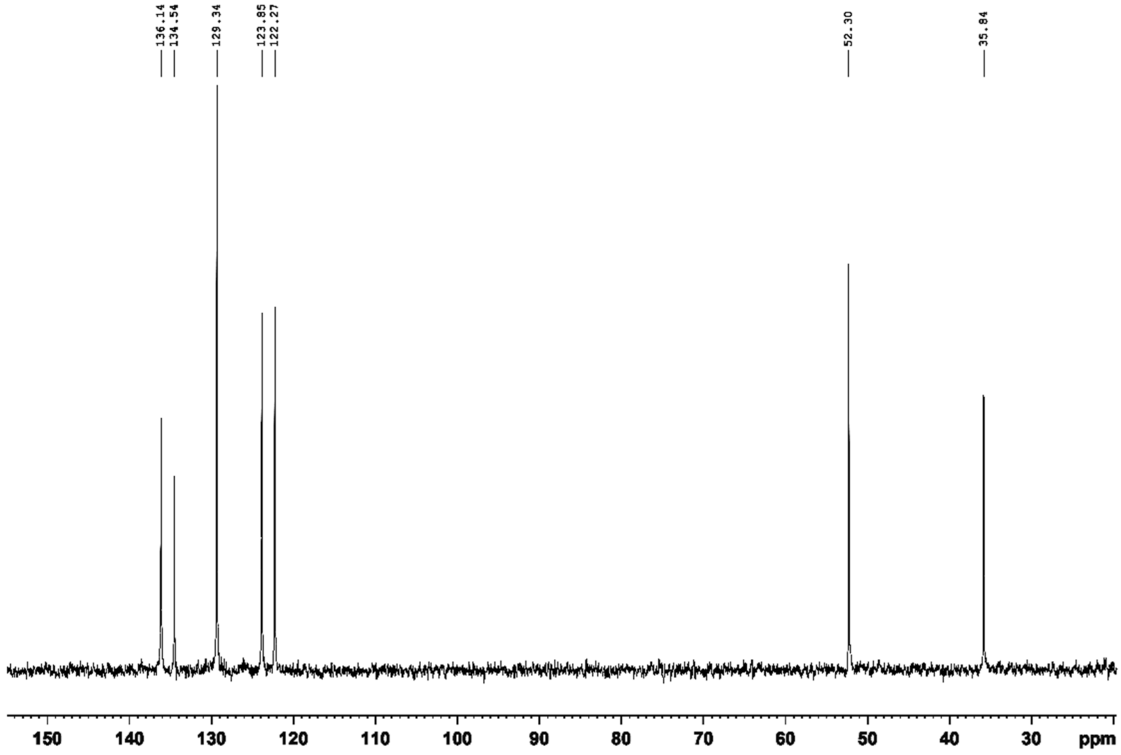

Figure S12. ^{13}C-NMR spectrum of 1,1'-(1,4-phenylenebis(methylene))bis(3-methylimidazolium) tungstate (**6**).

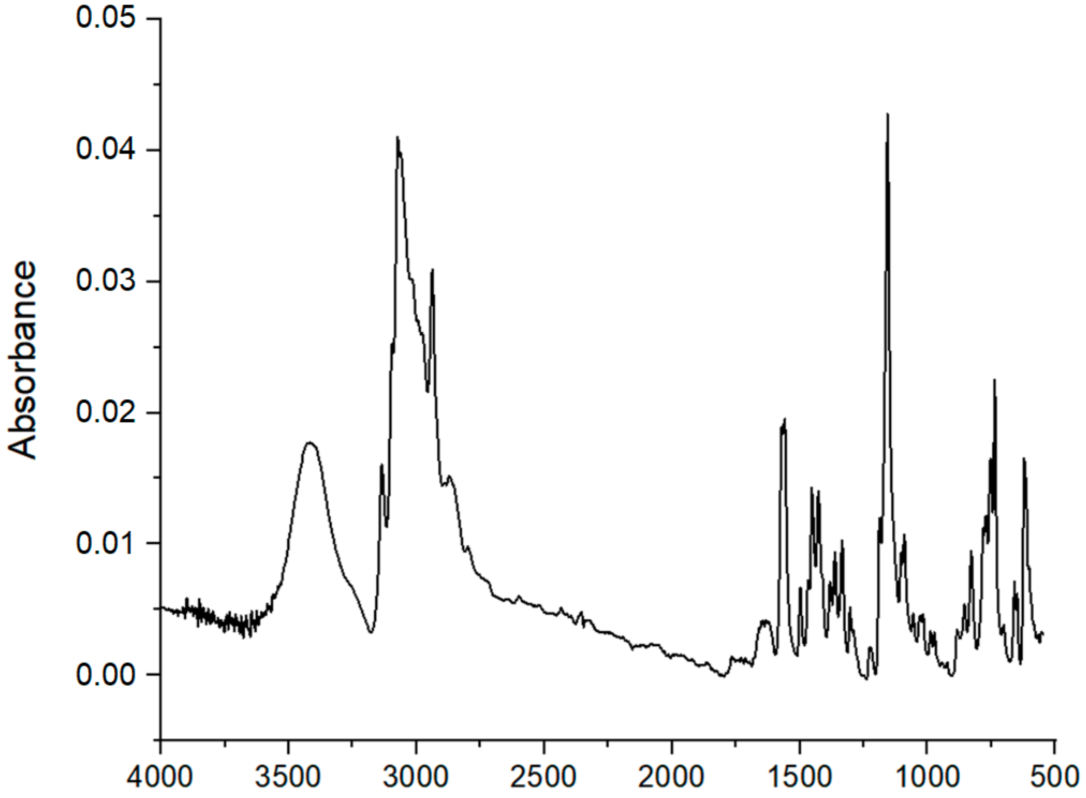

Figure S13. ATR-FTIR spectrum of 1,1'-(1,2-phenylenebis(methylene))bis(3-methylimidazolium) bromide (**1**).

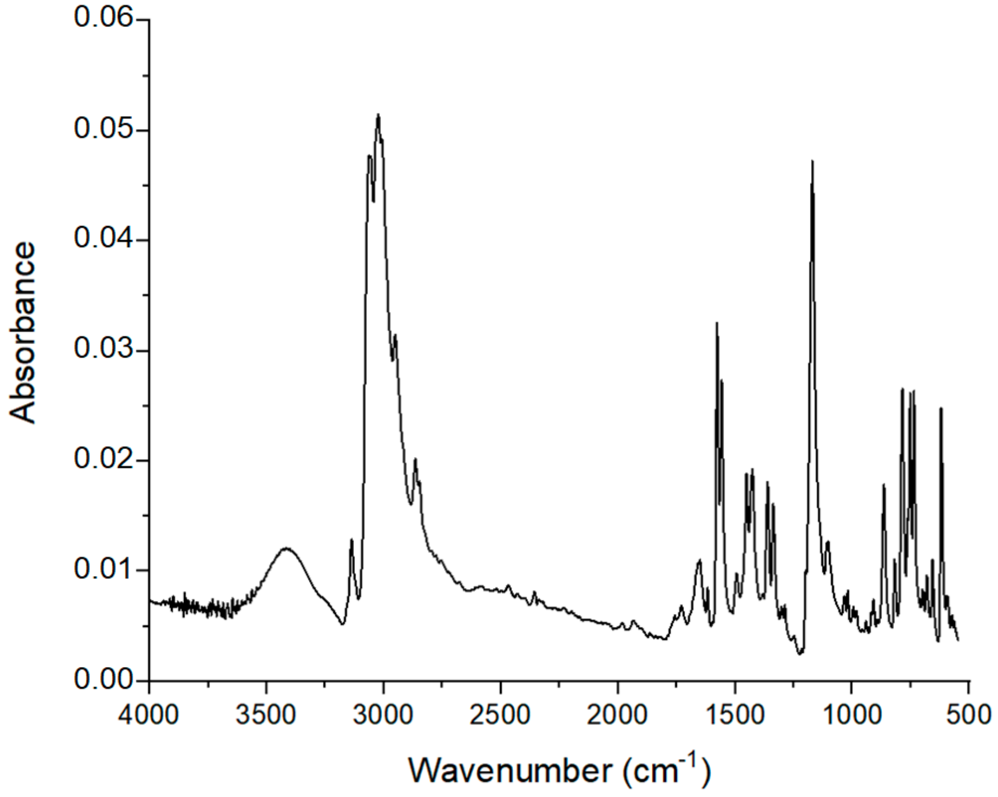

Figure S14. ATR-FTIR spectrum of 1,1'-(1,3-phenylenebis(methylene))bis(3-methylimidazolium) bromide (**2**).

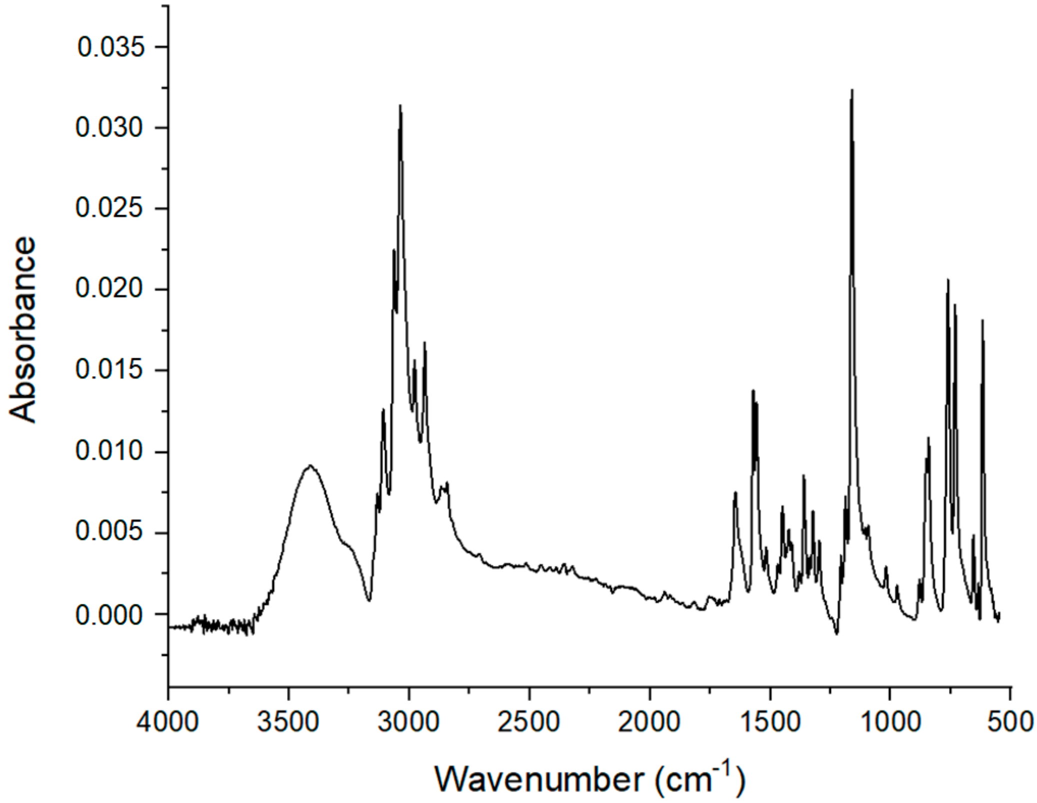

Figure S15. ATR-FTIR spectrum of 1,1'-(1,4-phenylenebis(methylene))bis(3-methylimidazolium) bromide (**3**).

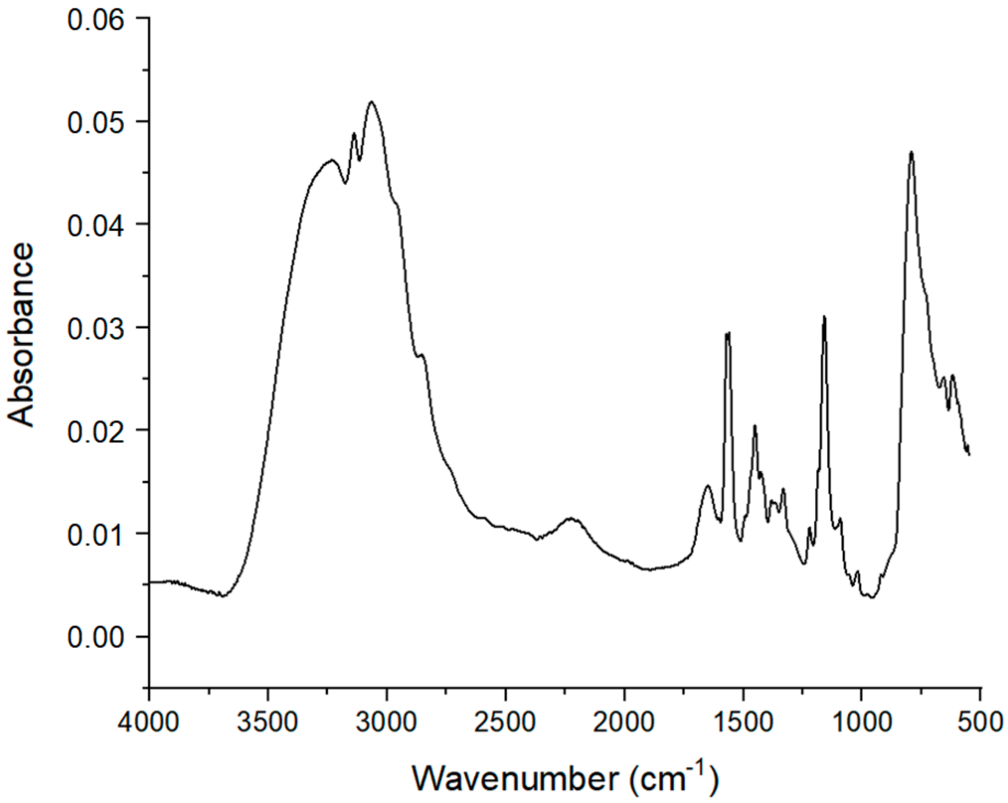

Figure S16. ATR-FTIR spectrum of 1,1'-(1,2-phenylenebis(methylene))bis(3-methylimidazolium) tungstate (**4**).

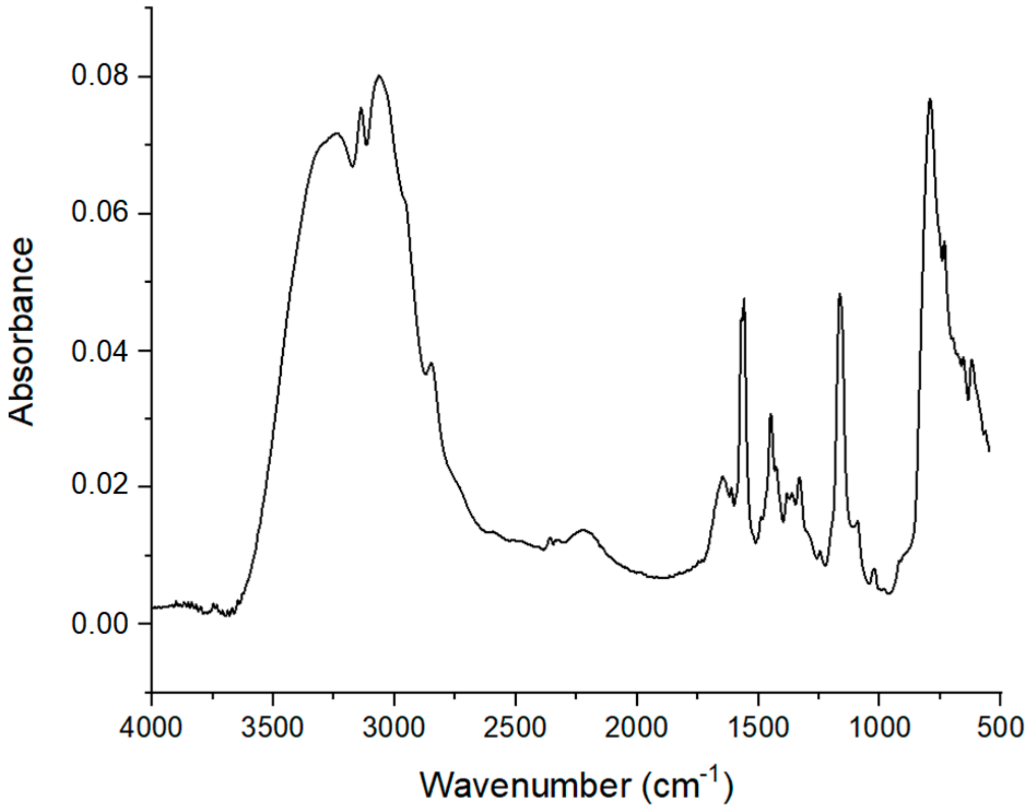

Figure S17. ATR-FTIR spectrum of 1,1'-(1,3-phenylenebis(methylene))bis(3-methylimidazolium) tungstate (**5**).

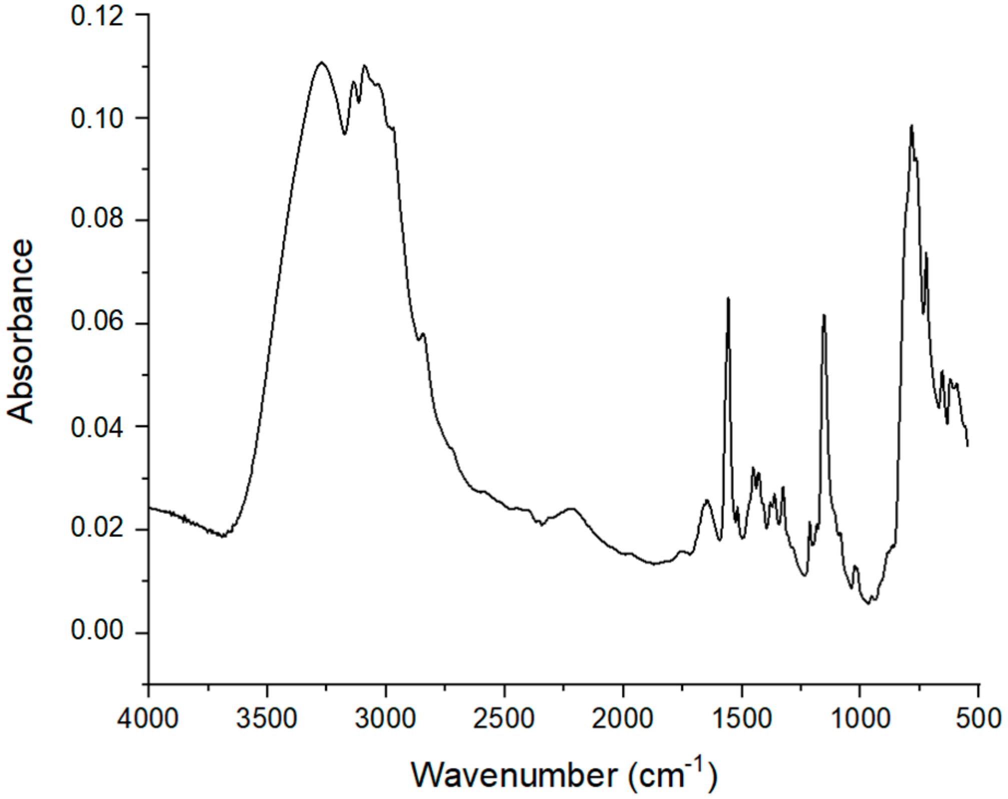

Figure S18. ATR-FTIR spectrum of 1,1'-(1,4-phenylenebis(methylene))bis(3-methylimidazolium) tungstate (**6**).

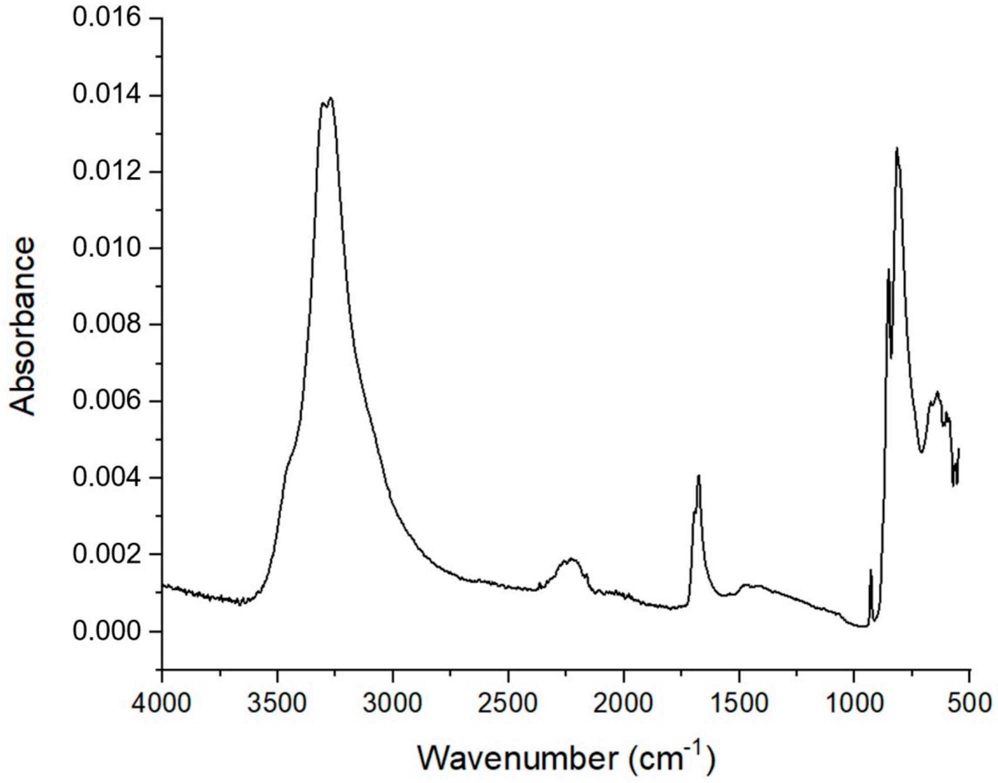

Figure S19. ATR-FTIR spectrum of sodium tungstate.

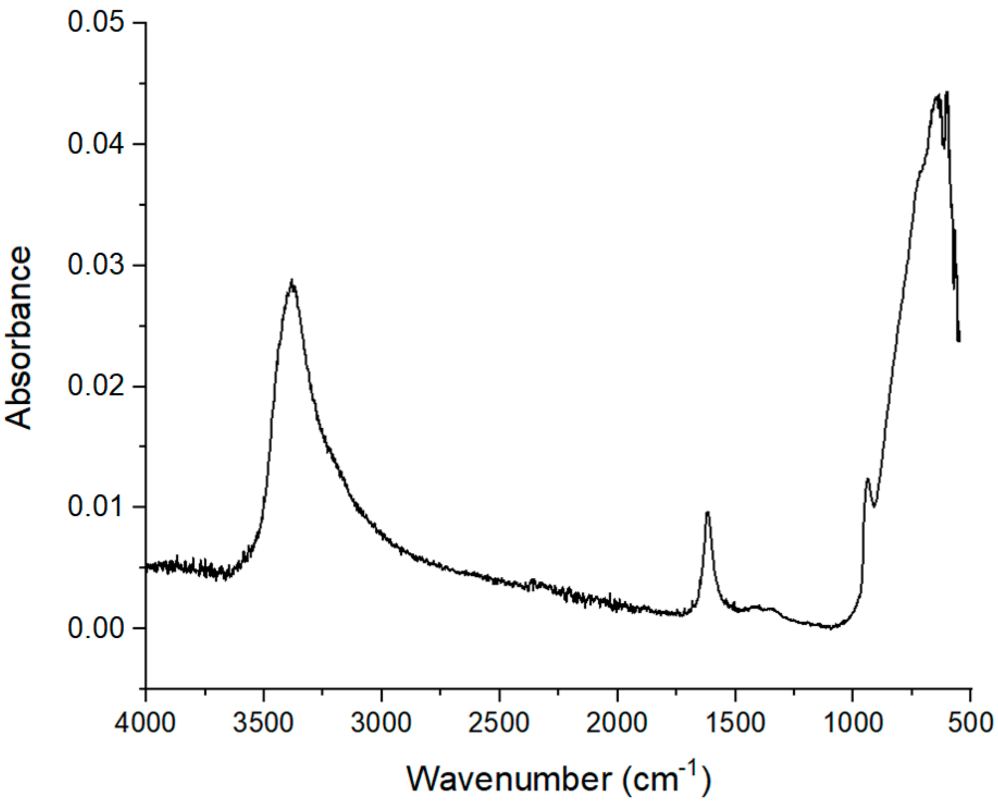

Figure S20. ATR-FTIR spectrum of tungstic acid.

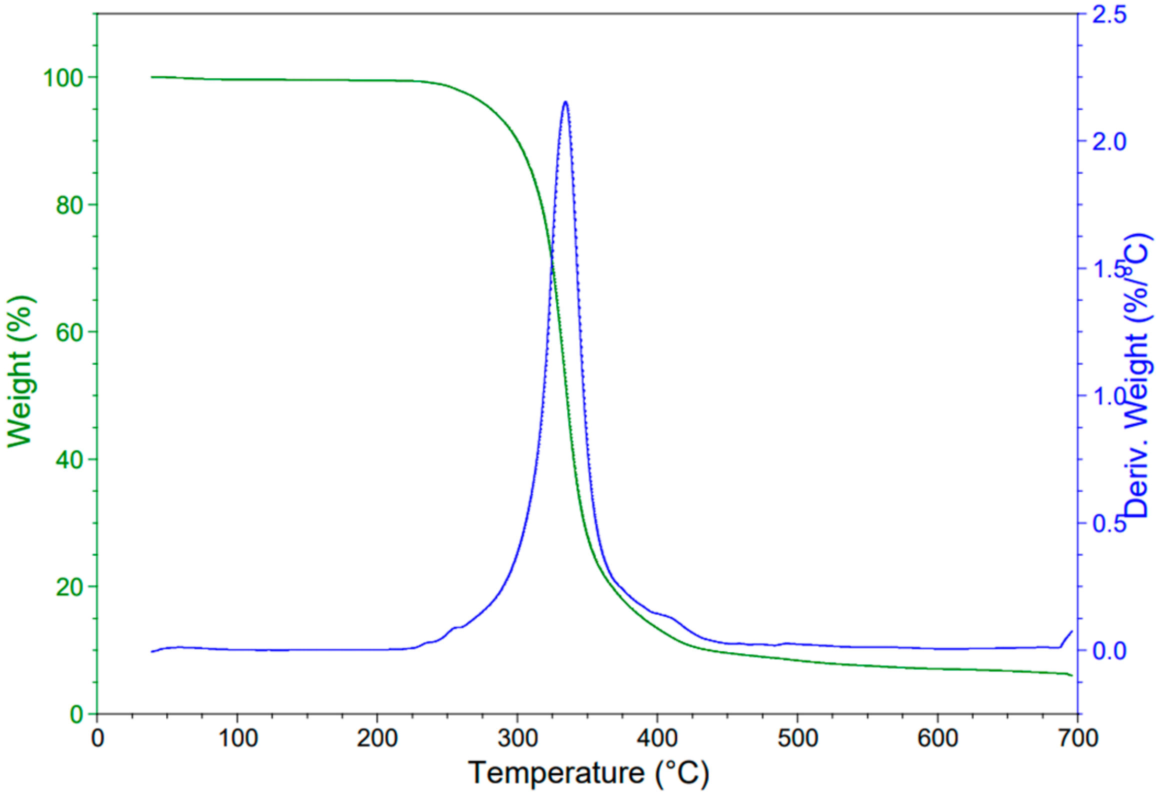

Figure S21. Thermal gravimetric analysis and derivative of 1,1'-(1,2-phenylenebis(methylene))bis(3-methylimidazolium) bromide (**1**)

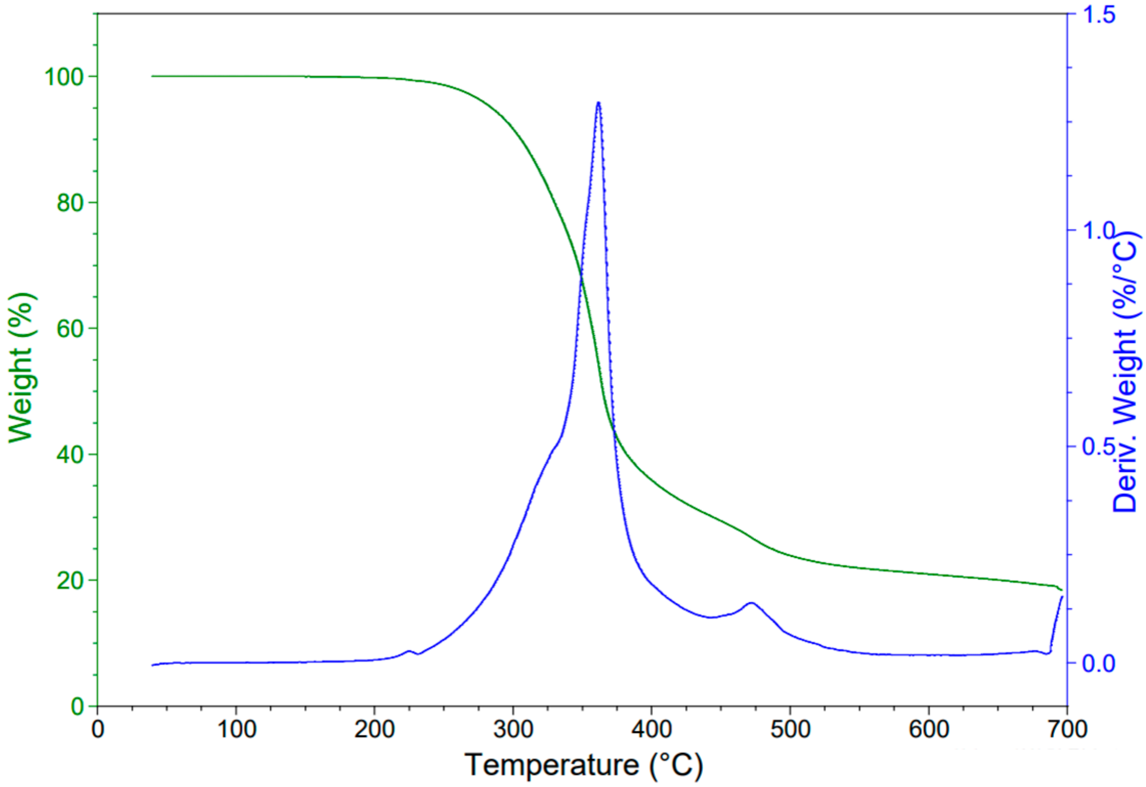

Figure S22. Thermal gravimetric analysis and derivative of 1,1'-(1,3-phenylenebis(methylene))bis(3-methylimidazolium) bromide (**2**)

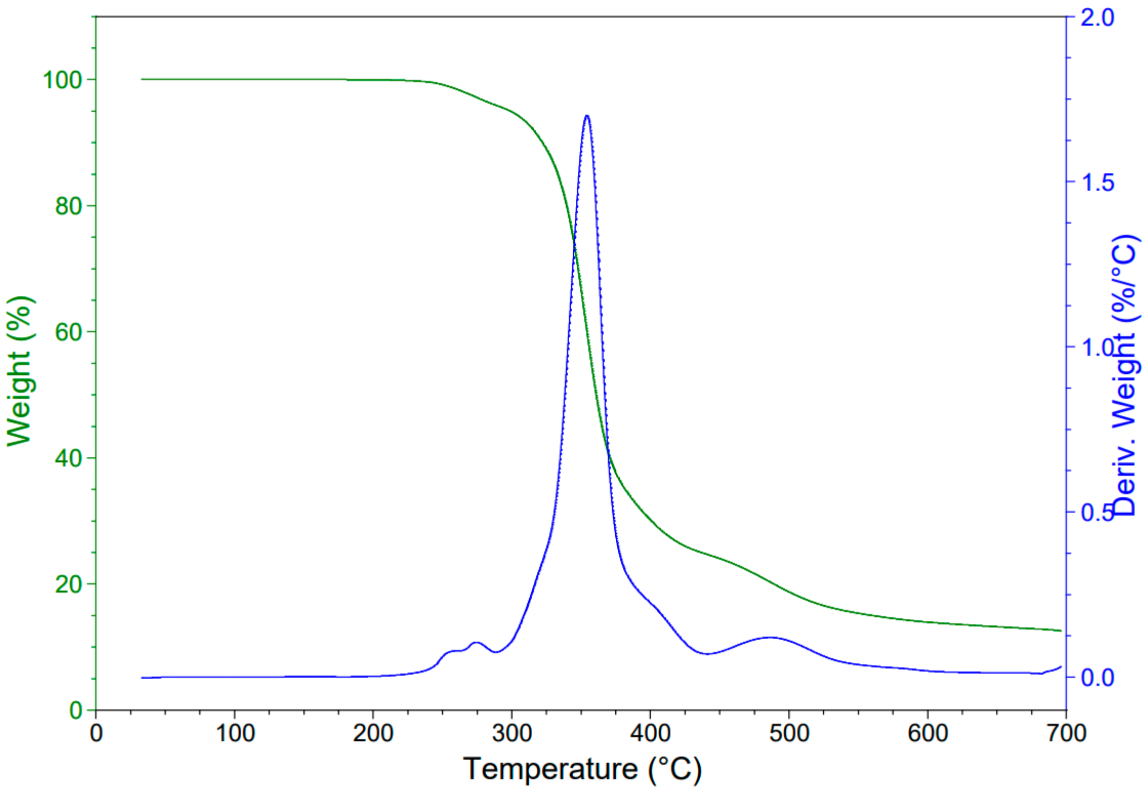

Figure S23. Thermal gravimetric analysis and derivative of 1,1'-(1,4-phenylenebis(methylene))bis(3-methylimidazolium) bromide (**3**)

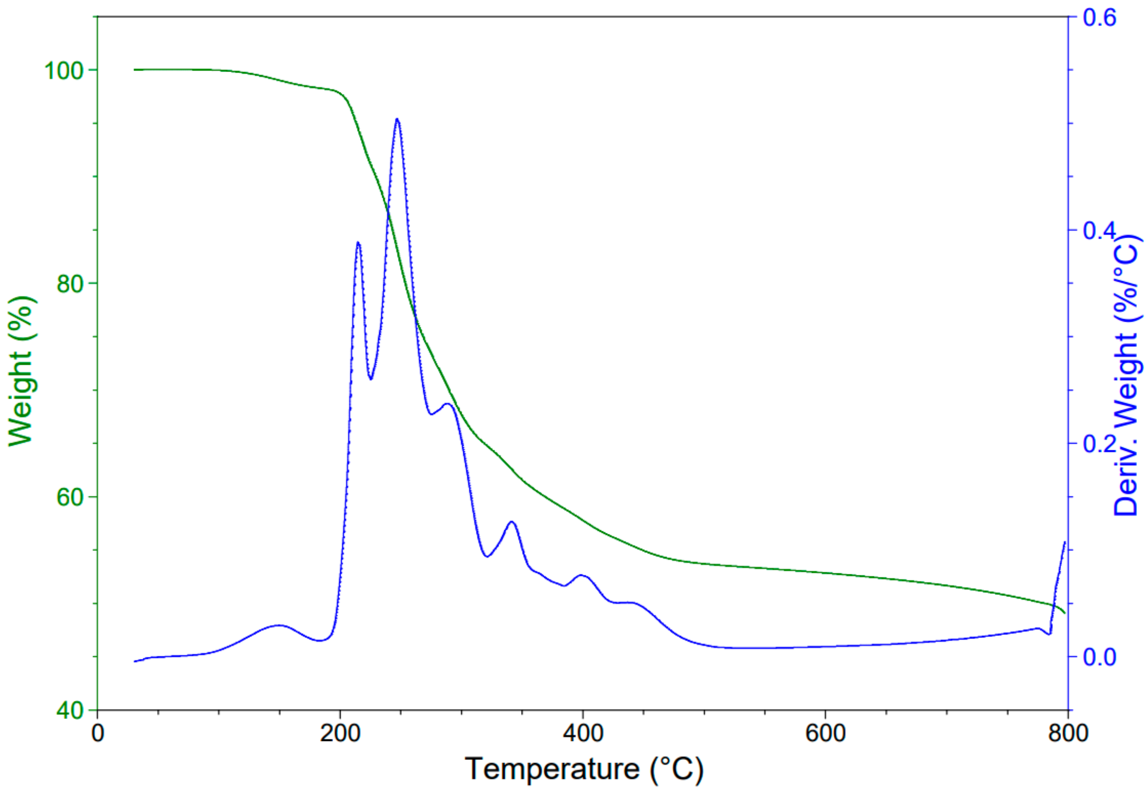

Figure S24. Thermal gravimetric analysis and derivative of 1,1'-(1,2-phenylenebis(methylene))bis(3-methylimidazolium) tungstate (**4**).

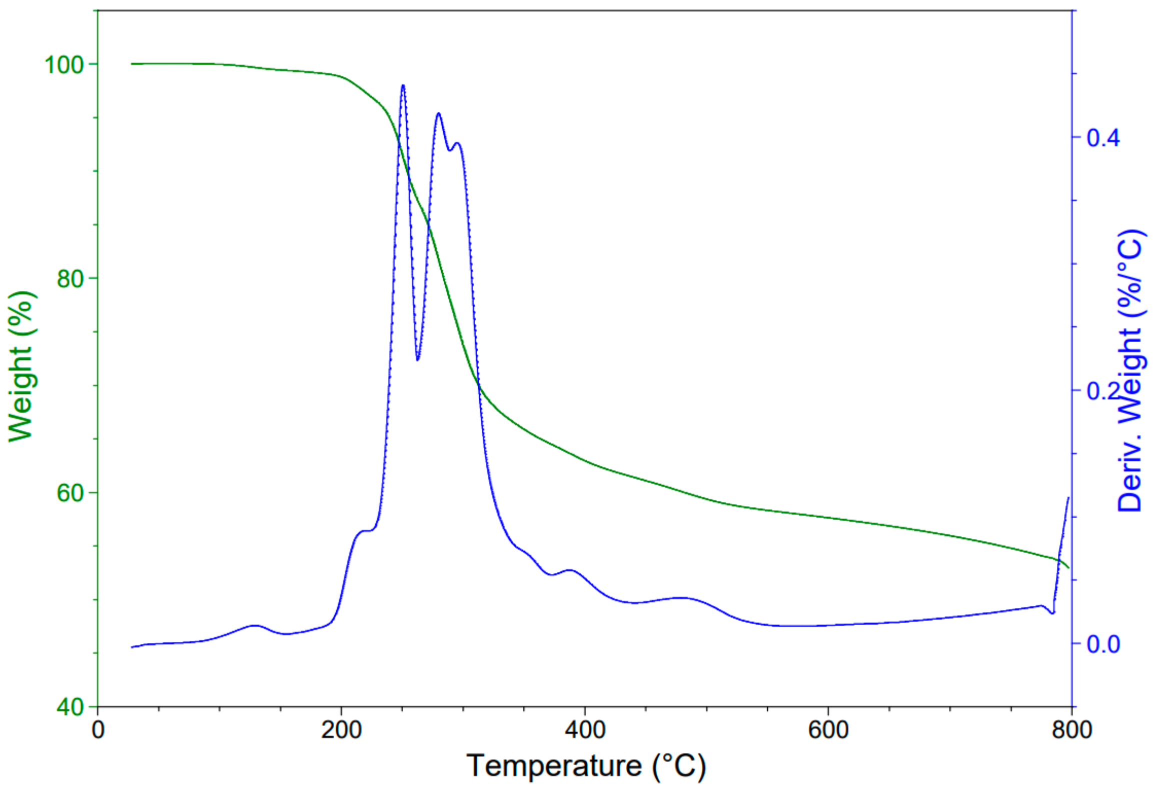

Figure S25. Thermal gravimetric analysis and derivative of 1,1'-(1,3-phenylenebis(methylene))bis(3-methylimidazolium) tungstate (**5**).

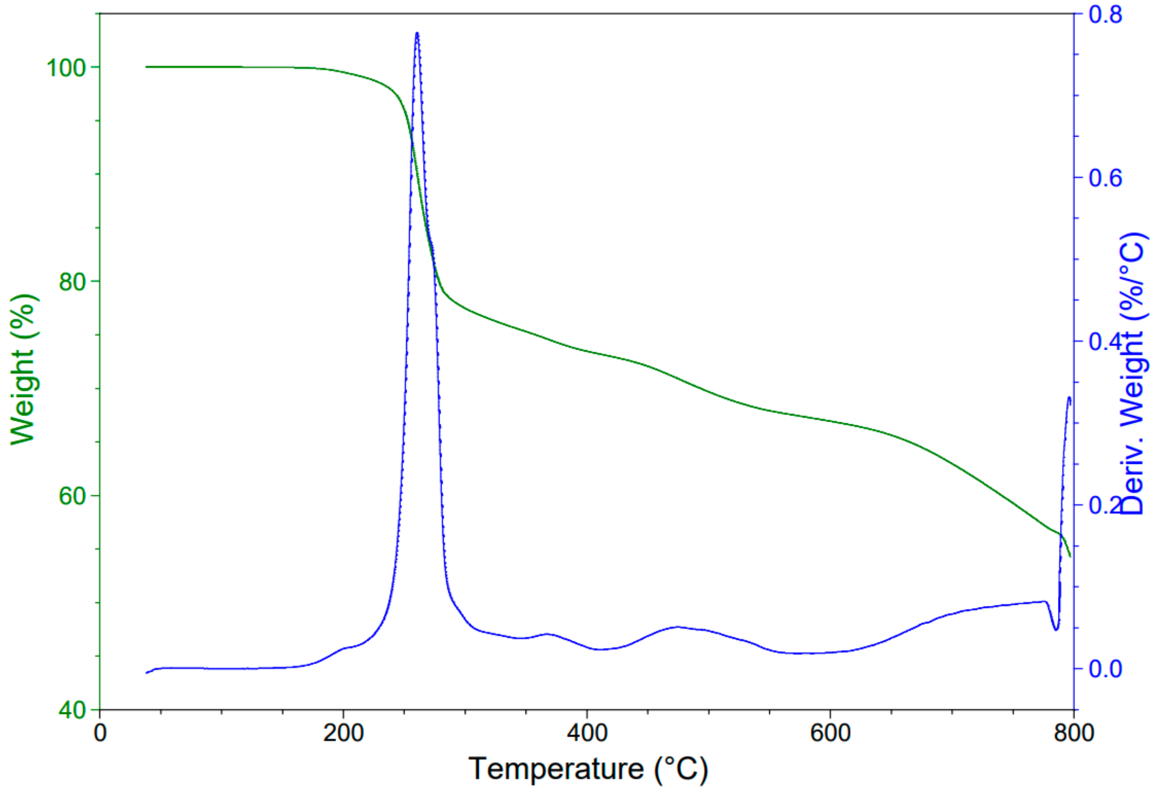

Figure S26. Thermal gravimetric analysis and derivative of 1,1'-(1,4-phenylenebis(methylene))bis(3-methylimidazolium) tungstate (**6**).

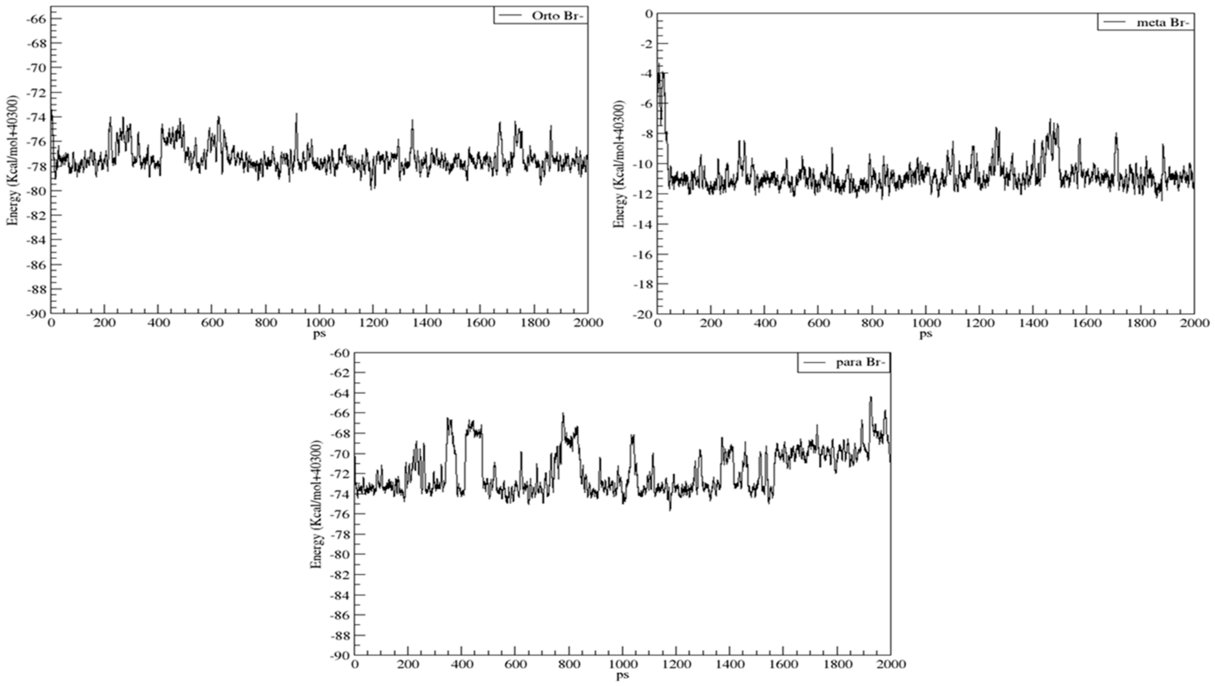

Figure S27: Energy variation along the simulation time to the IL using the Bromide anion (Conformations Ortho, Meta and Para).

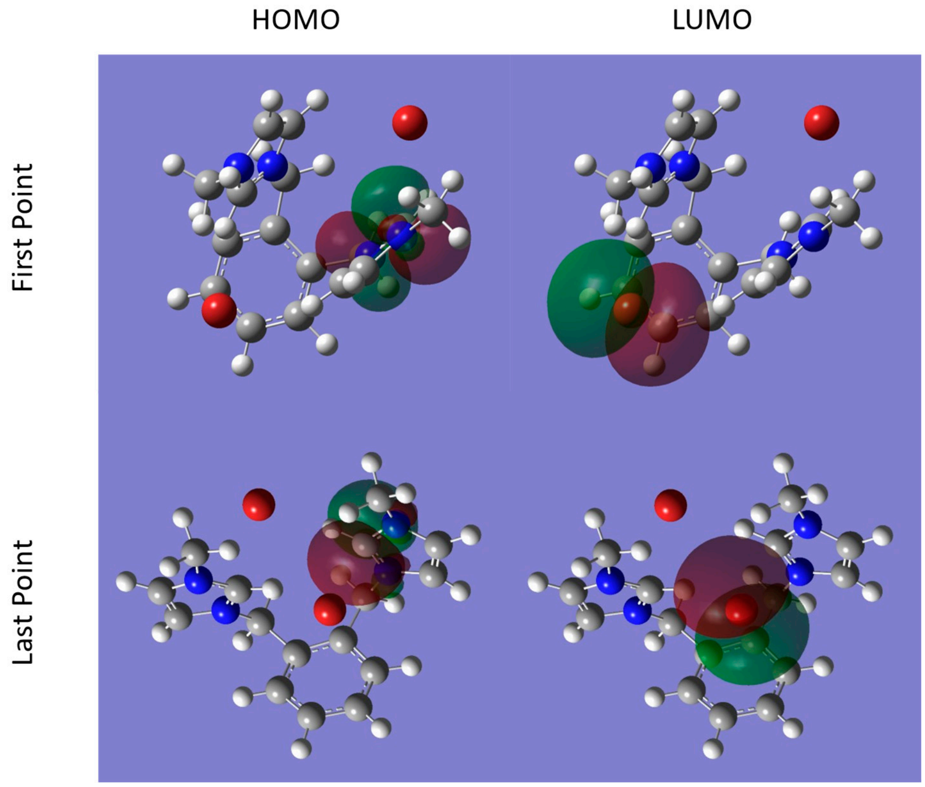

Figure S28: HOMO-LUMO analyses for the IL with bromide anion in the configuration ortho.

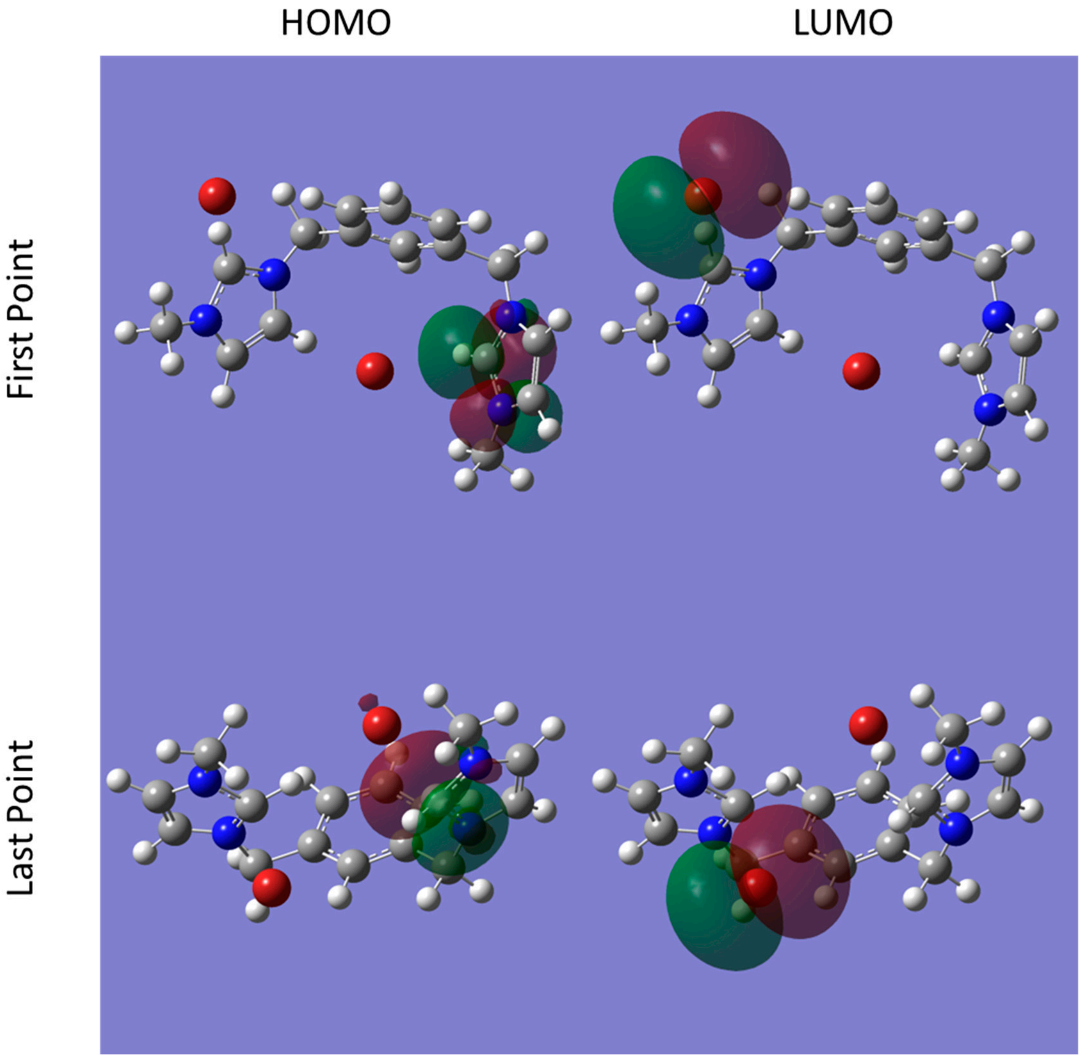

Figure S29: HOMO-LUMO analyses for the IL with bromide anion in the configuration meta.

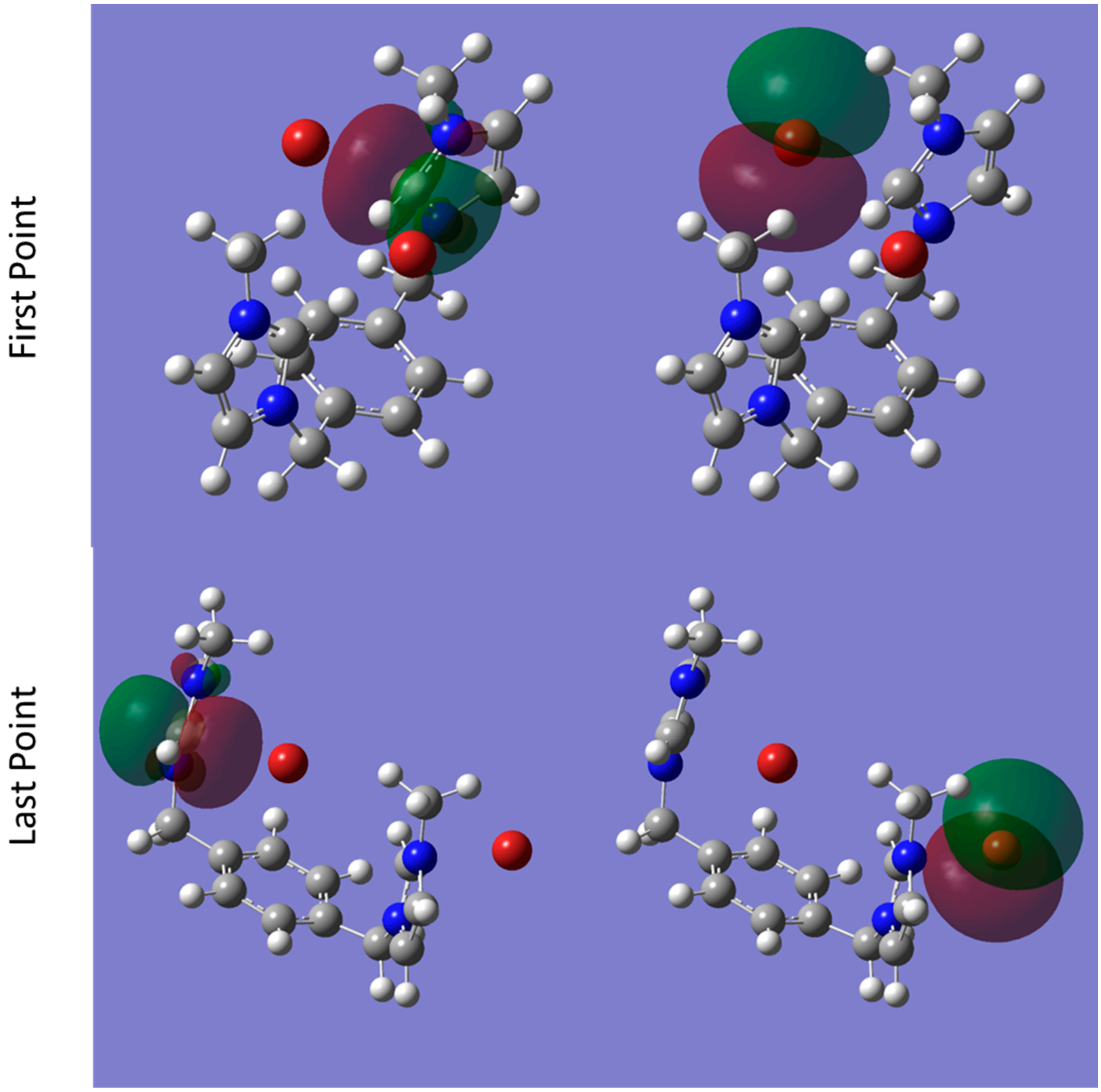

Figure S30: HOMO-LUMO analyses for the IL with bromide anion in the configuration para.

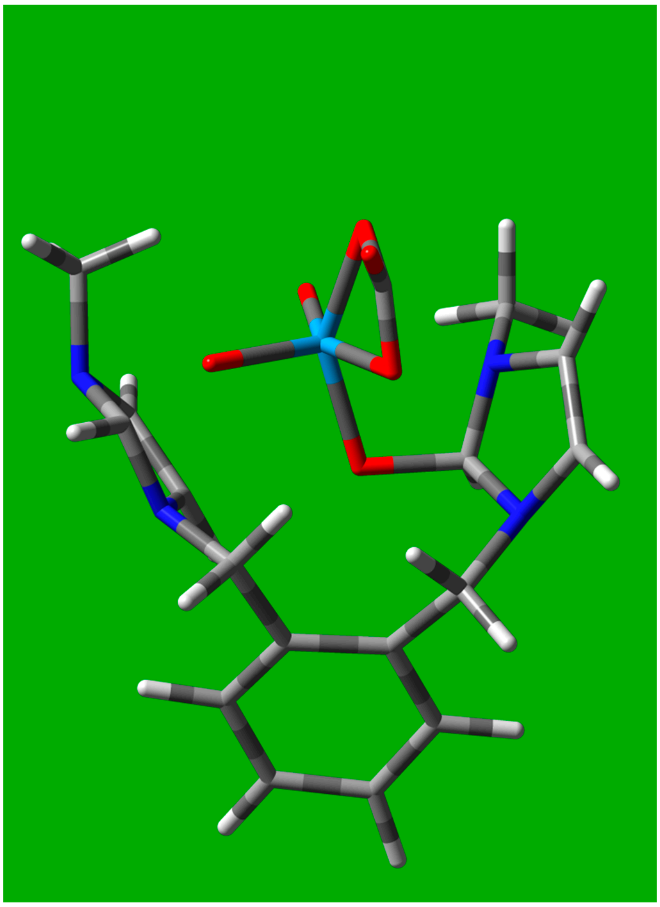

Figure S31. Structure of cluster with CO_2. Ortho configuration.

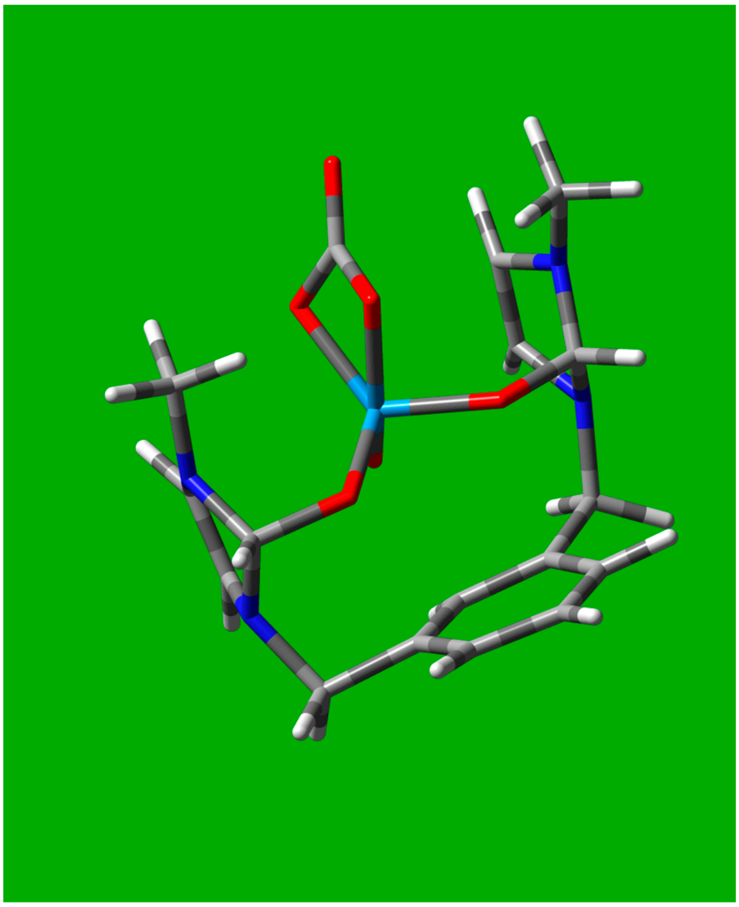

Figure S32. Structure of cluster with CO_2. Meta configuration.

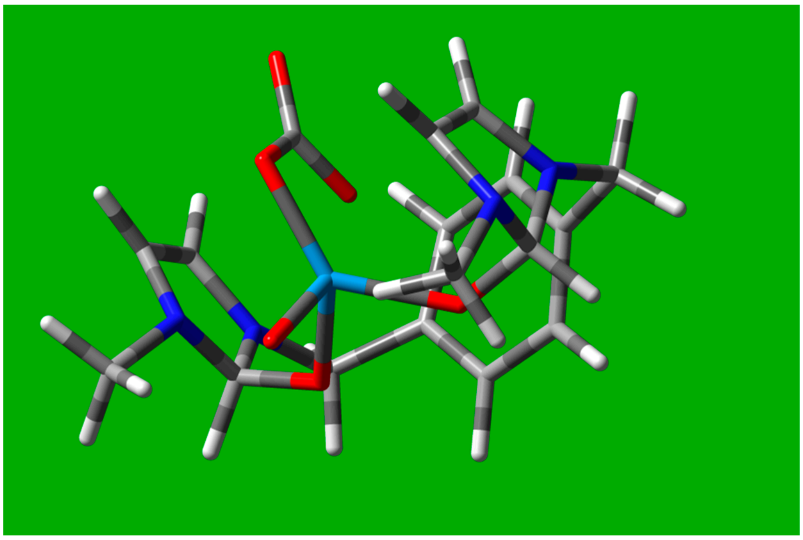

Figure S33. Structure of cluster with CO_2. Para configuration.
